# ROS-Mediated Enamel Formation Disturbance Characterized by Alternative Cervical Loop Cell Proliferation and Downregulation of RhoA/ROCK in Ameloblasts

**DOI:** 10.1155/2022/5769679

**Published:** 2022-10-17

**Authors:** Yuchan Xu, Yunyan Zhang, Jingwen Zheng, Mingxue Xu, Yuzhi Yang, Weihua Guo

**Affiliations:** ^1^State Key Laboratory of Oral Diseases, West China Hospital of Stomatology, Sichuan University, 610041 Chengdu, China; ^2^National Clinical Research Center for Oral Diseases, West China Hospital of Stomatology, Sichuan University, 610041 Chengdu, China; ^3^Department of Pediatric Dentistry, West China School of Stomatology, Sichuan University, Chengdu 610041, China

## Abstract

Reactive oxygen stress (ROS) is generally accepted as a signal transducer for coordinating the growth and differentiation of tissues and organs in the oral and maxillofacial region. Although ROS has been confirmed to affect the development of enamel, it is not yet known that the specific mechanism of ROS accumulation induced enamel defects. Given the lack of knowledge of the role of ROS in enamel, the aim of the study is to determine how oxidative stress affects cervical cells and ameloblast cells. Using SOD1 knockout mice, we identified a relationship between ROS fluctuations and abnormal enamel structure with HE staining, micro-CT, and scanning electron microscope. Increased ROS induced by H_2_O_2_, certified by the DCFH probe, has resulted in a dual effect on the proliferation and differentiation of cervical cells, indicating a higher tendency to proliferate at low ROS concentrations. Ameloblasts transfected with SOD1 siRNA showed a significant reduction of RhoA and ROCK. This study investigates for the first time that SOD1-mediated ROS accumulation disrupted normal enamel structure through alternative cervical loop cell proliferation and downregulation of RhoA and ROCK in ameloblasts, demonstrating the convoluted role of ROS in monitoring the progress of enamel defects.

## 1. Introduction

Oxidative stress is the unbalanced state of ROS and antioxidants, which leads to tissue damage or cellular injury [1]. ROS regulates growth, differentiation, adhesion, aging, and apoptosis of cells as signaling molecules at low concentrations, whereas ROS turns to be harmful to cells at high concentrations through oxidation of cellular proteins and lipids, damage to DNA, and aberrant cell signaling pathways [2]. Among the antioxidant enzyme system and antioxidant agents, superoxide dismutase (SOD) alleviates oxidative intermediate accumulation most effectively via catalyzing the dismutation of superoxide anions [3]. As the most studied ROS scavenging member of superoxide dismutase, SOD1 has also been identified as a controversial participant in promoting certain cancer types recently. The noncanonical role of SOD1 was even presented as a complex regulator of transcription process and mitochondrial metabolism [4]. Since SOD1 mutation correlates to neuron motor degeneration-related diseases such as amyotrophic lateral sclerosis (ALS), the SOD1-deficient mouse model now is recognized as a classic study model for ALS [3]. Furthermore, such mouse model has been confirmed as an effective tool to explore the oxidation microenvironment of physiologic development as well as pathological diseases in other fields like bone [5–7].

The stem cells residing in the incisor cervical loop act as the dental epithelial progenitor cells and allow rodent incisors to continue to erupt continuously, serving as an excellent model for tooth development [8]. Mainly located at the labial incisor apex, cervical loop stem cells bring about inner enamel epithelial cells, which then give rise to ameloblasts, while generate identical cells to reserve the stem cell pool in the niche [9]. The fundamental molecular networks in the mesenchyme regulate the division of stem cells and determination of ameloblasts, fine-tuned by positive regulators including fibroblast growth factor (FGF) pathway and specific inhibitors encompassing BMP4 and Wnt/*β*-catenin [10–12]. In line with other renewal tissues, the cervical loop stem cells accomplish a dynamic balance between proliferation and differentiation with delicate modulation signals [13, 14].

Differentiated by inner enamel cells, ameloblasts experience three different stages including preameloblasts, secretory ameloblasts, and maturation ameloblasts, which characterized enormous changes in specific cell polarity. During such process, the morphology of highly polarized ameloblasts turns to be tall columnar, the nucleus is far away from the basement membrane, and cell organelles like mitochondria and Golgi apparatus locate at near or away from the basement membrane, respectively. Several signals and pathways participate in the regulation of ameloblast polarity formation involving Rho GTPase, planar cell polarity (PCP) proteins, metalloproteinase 20 (MMP20), special AT-rich sequence-binding protein 1 (SATB1), and especially Wnt-related pathways [15–19]. RhoA reduction was reduced either by RhoA knockout transgenic mice or by NaF treatment, and ameloblast lineage cell line activated canonical and noncanonical Wnt pathways resulting in enamel defects in mice [20]. Localized at the special Tomes processes in secretory ameloblasts, VANGL2, which is the core PCP protein, determines secretory surfaces and stable adherence junctions [21]. As an enzyme resulting in enamel matrix protein cleavage, MMP20 participates into amelogenesis at the proper expression level while the overexpression leads to increase of inactive p-cofilin, disruption of normal polarity, and enhanced Wnt-mediated cell migration [18]. The Satb1-/- mice with deformed ameloblasts and hypomineralized enamel impair the structure of Tomes processes, cellular adhesion, and transportation of enamel matrix proteins [16].

As the earliest cloned proteins in the Ras superfamily, Rho GTPases are a group of guanosine triphosphate (GTP) binding proteins with a relative molecular mass of about 20~ 25 kD. Rho GTPase is active when binding to GTP while inactive when binding to GDP, acting like a biomolecular switch to regulate actin cytoskeleton [22]. The Rho family controls the pathways involved in various physiological processes such as cell proliferation, cell adhesion, and apoptosis by activating and stimulating downstream effectors. Among all 20 members of the Rho GTPase superfamily, RhoA, Rac1, and Cdc42 are the most studied ones. Participating in the formation of tension fibres and the assembly of focal adhesion complexes, RhoA and the downstream effector Rho-associated protein kinase 1 (ROCK1) promote myosin light chain (MLC) phosphorylation and inactivation of myosin light chain phosphatase (MLCP) [23, 24]. Furthermore, it is ascertainable that Sema4D-RhoA-AKT signaling pathway possesses ameloblast differentiation and amelogenin expression spatiotemporally [25]. Fibronectin/laminin-integrin-odontogenic ameloblast-associated protein- (ODAM-) Rho guanine nucleotide exchange factor 5- (ARHGEF5-) RhoA pathway has been identified in junctional epithelium (JE) development and regeneration process to maintain periodontal health [26]. Upregulation of Wnt pathway activity caused by inhibition of RhoA disrupted ameloblast differentiation, which confirmed a crosstalk between Wnt and RhoA existed in enamel fluorosis [27]. As mediators of both tight junction and cytoskeleton, RhoA plays an essential role in ameloblast development.

## 2. Materials and Methods

Materials and Methods should contain sufficient detail so that all procedures can be repeated. It may be divided into headed subsections if several methods are described. In ethical statement, all animal experiments and procedures in this study were in accordance with the principles and procedures of institutional guidelines on laboratory animal welfare and were approved by the Ethics Committee of the State Key Laboratory of Oral Diseases, West China Hospital of Stomatology (Chengdu, Sichuan) (permit no. WCHSIRB-D-2021-053).

### 2.1. Animals, Genotyping, and Grouping

SOD1 (-/-), SOD1 (+/-), and SOD1 (+/+) mice were generated by mating congenic B6 < Sod1 +/->. 129S-Sod1tm1Leb/J which were purchased from Shanghai Southern Model Biotechnology Co., Ltd., breed in a pathogen-free facility at Sichuan University Laboratory Animal Centre and feed with SPF animal feed and drinking water. Lighting follows a 12 : 12 circadian rhythm. Light intensity is 130-325 lux, relative humidity was 40-60%, noise was 25-40 dB, room temperature was 21 ± 3°C, and ventilation was 10-15 times/hour. SOD1 (+/-) mice were selected for colony reproduction and maintenance since female infertility in SOD1 (-/-) mice. Select SOD1 (+/-) knockout mice older than 8 weeks as breeders: 1♂×2♀ per cage to obtain SOD1 (-/-) (homozygotes), SOD1 (+/-) (heterozygotes), and SOD1 (+/+) (wild type). Evaluate the genotype of each mouse by quantitative PCR of DNA isolated from tail biopsy samples, as described on the Jackson Laboratory website. 8 cages were used for breeding with the ratio of female and male of 1 : 3 and 3 cages for pregnant mice, ensuring that the pregnant mice were isolated separately. The female donor mice were sacrificed after 5-6 births, and male donor mice were sacrificed after 7 months of age. The SOD1 homozygous mouse (SOD1 (-/-)) was used as the experimental group, the littermate SOD1 wild-type mice (SOD1 (+/+)) were used as the control group, and the SOD1 heterozygous mice (SOD1 (+/-)) were used as the breeding group. During the breeding process, it was not found that SOD1 gene knockout had a significant effect on the early survival of mice.

### 2.2. Tissue Preparation and HE Staining, Immunofluorescence Staining, and Immunohistochemical Staining

Harvest the mandibles from 3 mice each in the WT group and SOD1 knockout group, fix in 4% paraformaldehyde overnight at 4°C, decalcify with 10% EDTA at 37°C for 4 months, then dehydrate in ethanol, embed in paraffin wax, and serially section at 5 *μ*m. Sections were stained with hematoxylin and eosin (H&E) according to the manufacturer's recommended protocol. The IF staining was performed on tissue sections. The primary and secondary antibodies and their dilutions used in this study were as follows: Rho-A (1 : 100, Abcam, USA), AMBN (1 : 50, Santa Cruz, USA), AMGN (1 : 50, Santa Cruz, USA), Alexa Fluor 488 goat anti-rabbit (1 : 1000, Invitrogen, USA), and Alexa Fluor 488 goat anti-mouse (1 : 1000, Invitrogen, USA). The images were observed and taken under microscope (Olympus, Japan). Permeabilized with 0.25% Triton-X 10 min, 3% H_2_O_2_ was dropped onto the slides to remove endogenous peroxidase at room temperature for 10 minutes, blocked for 30 min with 5% bovine serum albumin (BSA) at room temperature, and subsequently incubated with primary antibodies overnight at 4°C. Incubating the specimens with the secondary antibody according to manufacturer's protocol, 1% BSA was used in place of the primary antibodies as the negative control, and secondary antibodies were visualized using the DAB kit (Gene Tech, China). Antibodies included Ki67 (Abcam, USA), AMBN (Santa Cruz, USA), and AMGN (Santa Cruz, USA) at a dilution of 1 : 200.

### 2.3. Microcomputerized Tomography (Micro-CT)

Three left mandibles of both groups were performed for micro-CT scanning. Choose a 15 mm scanning tube, fix the processed experimental specimen in the scanning tube, and place specimen into the micro-CT machine, ensuring the sagittal plane of the mandibles perpendicular to the bottom surface of the scanning tube. The scanning range included the mandible and its extension 0.5 mm up and down. The scanning parameters are X-ray tube voltage 90 kV, current 200 *μ*A, resolution 9 *μ*m, and exposure time 500 ms. Perform three-dimensional reconstruction analysis of the mandible in the micro-CT supporting software. For each sample, the enamel part of the mandibular incisor was circled, and the density threshold was above 1500 mg/HA ccm to calculate the average density of the mouse mandibular incisor.

### 2.4. Scanning Electron Microscope

Take randomly 3 mice each in the control group and the experimental group of 4 months old and cut off the end of their lower incisor, about 2-3 mm as the sample. Wash with normal saline twice, blow dry, and etch the enamel surface with 30% phosphoric acid for about 45-60 seconds. Put them into 0.6 mL EP tubes, number them, add distilled water, and put them in an ultrasonic oscillator for 5-6 times, 30 minutes each time. Replace the distilled water in the EP tube every time.

### 2.5. Acquisition and Cell Culture of Incisor Cervical Loop Stem Cells and HAT7 Cells

SD rats aged 5 days after birth were sacrificed by cervical dislocation under overdose anesthesia. The mandible was dissected, and then, the incisors were separated with the help of a stereomicroscope under aseptic conditions with microforceps. Dissect the labial end tissue of the incisor germ to fully reveal the cervical loop. The thin layer of the tooth sac above the incisor germ was gently separated, and the cervical loop was separated from the incisor germ with a blade. Then, the tissue was cut into small pieces by ophthalmic scissors and digested with 625 U/mL type I collagenase and 2.4 U/mL Dispase II at 37°C for 30 minutes. Shake the mixture every 10 minutes during the digestion. After digestion, it was centrifuged at 1200 rpm for 5 minutes and then cultured them in epithelial cell culture medium (Sciencell, 4101). The medium consists of basal medium and 2% fetal bovine serum (FBS), 1% epithelial growth factor, and 1% antibiotic solution. Incubate the cells at 37°C in a humid environment containing 5% CO_2_. After the cells grew and fused 80%, trypsin was used for differential digestion to purify the epithelial cells. The HAT-7 cell line was a gift from Professor Hidemitsu Harada of the Faculty of Dentistry, Osaka University Graduate School of Dentistry, Osaka, Japan. HAT-7 cells were seeded with Dulbecco Modified Eagle/F-12 (DMEM/F-12; Gibco-BRL, Grand Island, NY, USA) containing 10% FBS and penicillin-streptomycin at a humid atmosphere of 37°C and 5% CO_2_, and the medium was changed every 2 days. Prior to infection, cells were cultured in an antibiotic-free medium for 24 hours.

### 2.6. Identification of Cervical Loop Stem Cells and HAT7 Cells

The cells were fixed with paraformaldehyde, blocked with 1% BSA, incubated with primary antibodies overnight at 4°C, reacted with the corresponding secondary antibodies for 2 hours at 37°C the next day, then stained the nucleus with 40,6-diamino-2-phenylindole (DAPI; Sigma-Aldrich) for 5 minutes, and observed using the confocal fluorescence microscope. Antibodies used included CK14 (1 : 200, ab49747, Abcam), vimentin (1 : 200, OMA1-06001, Thermo), Sox2 (1 : 200, ab97959, Abcam), AMBN (1 : 200, sc50534, Santa Cruz), and AMGN (1: 200, sc32892, Santa Cruz).

### 2.7. siRNA-SOD1 Infection

The siRNAs were produced by GenePharma Co. Limited (Shanghai, China). The day before infection, cells were separated with trypsin and seeded in 6-well plates at 1.5 × 10^5^ cells/cm^2^. When grown to 80% confluence in 2 mL Dulbecco's Modified Eagle's Medium/F12 (DMEM/F12) in each well, the HAT7 cells were infected with a mixture of 150 pmol SOD1-siRNA and 8 *μ*L transmate each well without any serum. After 6 hours of transfection, the medium was replaced with DMEM/F12 containing 10% FBS. RNA-mediated degradation of the SOD1 expression was confirmed by quantitative reverse transcription polymerase chain reaction (RT-qPCR).

### 2.8. qPCR

24 hours after siRNA transfection, cells were harvested for RT-qPCR analysis, mRNA expression of SOD1, and RhoA pathway molecules (Table 1). Briefly, we used the FastPure Cell/Tissue Total RNA Isolation Kit V2 to extract total RNA and generate single-stranded cDNA. The expression levels of all target genes were calculated using the 2^-△△CT^ method and standardized to the level of the GAPDH expression.

Real-time quantitative polymer chain reactions (RT-qPCR) were performed with 10 ng cDNA. Real-time PCR was carried out using iTaqTM SYBR Green Supermix (Bio-Rad, Hercules, CA, USA) according to the manufacturer's instructions. 40 cycles and a hybrid temperature of 60°C (31 seconds) were used for the entire cDNA. All PCR reactions were performed in triplicate.

### 2.9. Western Blot

Digest cells to obtain the cell suspension, wash twice with precooled PBS and add with lysate (RIPA : PMSF : phosphatase inhibitor = 100 : 1 : 1) 4°C for 15 minutes. Centrifuge at 12000r/15 min at 4°C to obtain the supernatant. Use BCA assay to measure the protein concentration. Add loading buffer and denatured protein at 99°C for 10 minutes. Western blot was conducted as previously described. The primary and secondary antibodies and their dilutions were as follows: AKT (1 : 1000, #4685, CST), P-AKT (1 : 1000, #4060, CST), P21 (1 : 1000, Ab18209, Abcam), and GAPDH (1 : 5000, Zen, China) used as the internal control. WB signals were detected using ImageJ in triplicates.

### 2.10. Cell Proliferation

Use TrypLE Express to digest the second-generation stem cells for 15 minutes. 3000 cells/100 *μ*L per well were seeded in a 96-well plate. After 24 hours, the cells adhered to the wall. Mix 30% H_2_O_2_ with the epithelial cell culture medium to configure gradient concentration of H_2_O_2_. With 100 *μ*L per well, 5 replicate wells in each group, after 24 hours of treatment, were washed 3 times with PBS for 5 minutes each time. Mix CCK-8 solution and epithelial medium at a concentration of 1 : 10 and add 100 *μ*L of the mixture to each well. The culture plate was incubated for 2 hours in an incubator in the dark, and the corresponding absorbance value was measured at the dual wavelength of 450 nm. Cultivate for 7 consecutive days. Digest the second-generation stem cells with TrypLE Express for 15 minutes, then make a cell suspension at a concentration of 5 × 10^4^/mL, and inoculate 200 *μ*L per well into a confocal glass culture dish. The mixture of H_2_O_2_ and cell medium incubated cells for 6 hours. Add 500 *μ*L of 50 *μ*M EdU to each well for 2 hours. Fix cells with 4% paraformaldehyde for half an hour. Add 200 *μ*L of 1x Apollo reaction solution to each well for Apollo staining for half an hour. Add 200 *μ*L 1x Hoechst 33342 reaction solution to each well for DNA staining for half an hour. Observe and take pictures under a confocal fluorescence microscope.

### 2.11. DCFH-DA Probe

The cell suspension was made at a concentration of 5 × 10^4^/mL. 200 *μ*L per well was seeded into a confocal glass Petri dish, incubated for 6 hours, and rinsed with PBS twice for 5 minutes each time. Add 500 *μ*L of DCFH-DA (1 : 1000) diluted with medium to each well and incubate at 37°C in the dark for 30 minutes to load the probe. Rinse twice with warm PBS to wash off the unloaded probes and check the fluorescence intensity with a confocal fluorescence microscope.

### 2.12. Alizarin Red Staining

The cells were seeded in a 6-well plate at a density of1 × 10^5^ cells/well and cultured for 24 hours. After the cells were stretched to 80% confluence, they are stimulated with different concentrations of H_2_O_2_ medium for 24 hours, with 3 replicate wells in each group. Aspirate the medium after 24 hours, rinse with PBS twice, and change to the mineralization induction medium (consisting of DMEM +10% FBS and 3.0 mM calcium, 100 nM dexamethasone, 10 mM *β*-glycerophosphate, and 50 *μ*g/mL L-ascorbic acid composition). The mineralization induction medium was replaced every 3 days, and different gradient concentrations of peroxide were used to stimulate 12 hours before the replacement. After 15 days of continuous cultivation, stain with Alizarin Red and take pictures for observation.

### 2.13. Statistical Analysis

All experiments were performed independently at least three times. The statistical analysis was carried out in the SPSS 21.0 software using Student's *t*-test or by one-way analysis of variance (ANOVA). *P* < 0.05 was considered statistically significant.

## 3. Results

### 3.1. SOD1 Knockout Induces Hypoplasia of Incisor Enamel in Mice

**Figure 1:**
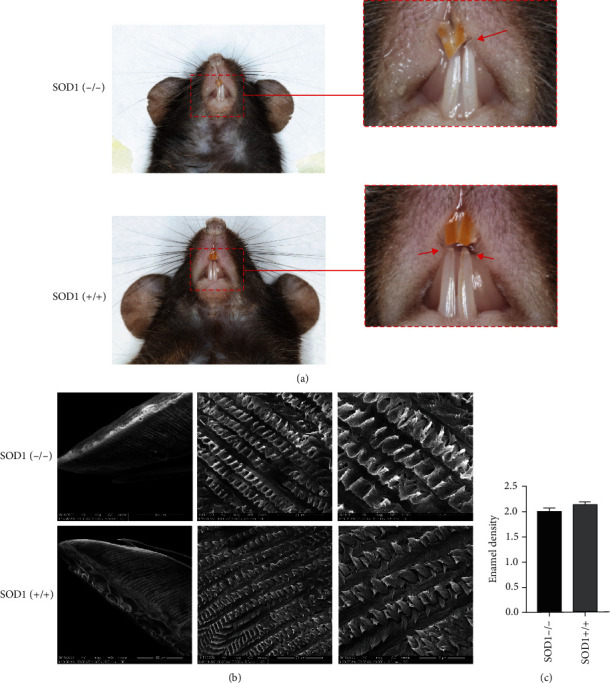
SOD1 knockout induces hypoplasia of incisor enamel in mice. (a) SOD1 (-/-) mice's incisor enamel is less translucent and has more abrasion in comparison to control mice. (b) The enamel rods of SOD1 (-/-) mice were loosely and porously arranged and lacked the traditional “braid-like” precisely staggered structure. (c) The difference of enamel mean density between SOD1 (-/-) mice and SOD1 (+/+) mice. *n* = 3, respectively.

In the control group (*n* = 3), the enamel length was normal, the enamel was translucent, the enamel surface was shiny, and the cut ends were sharp. However, in SOD1 gene knockout mice (*n* = 3), the enamel of the incisors obviously lost transparency, the cut ends were chalky, the color of the enamel surface disappeared, and the enamel surface was easily broken and showed irregular cross-sections (*n* = 3, Figure 1(a)). The enamel scanning electron microscope showed that the enamel microstructure of SOD1 knockout mice was aberrant. The enamel rods were arranged loosely, porously, and wavy and lost the normal “braid-like” tightly staggered structure (*n* = 3, Figure 1(b)). The micro-CT analysis also revealed that SOD1 knockout mice exhibit reduction of enamel mean density compared to the normal group (*n* = 3, respectively, Figure 1(c)).

### 3.2. ROS Accumulation Changed Cervical Loop Stem Cells as well as Ameloblast Structure with Decreased RhoA and AMGN Formation

**Figure 2:**
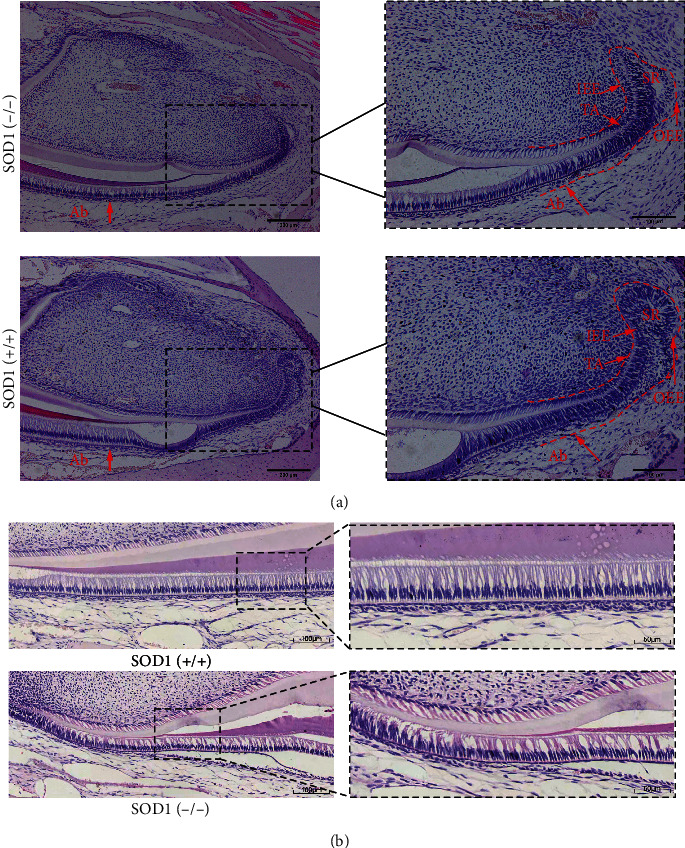
ROS accumulation changed cervical loop stem cells as well as ameloblast structures. (a) H&E staining of cervical loop cells. (b) H&E staining of ameloblasts. Scale bars: (a) 200 *μ*m and 100 *μ*m and (b) 100 *μ*m and 50 *μ*m.

The section of cervical loop revealed that the number of stellate reticulum layer cells shrinked, the outer enamel epithelial layer became thinner, and a clear gap was observed between the stellate reticulum cell layer and the outer enamel epithelial layer. The inner enamel epithelium and the metastatic proliferation zone were arranged disorderly (Figure 2(a)). Arrayed in high columnar shape, normal ameloblasts with palisade arrangement possess inverted polarity, whose nuclei are far away from the basement membrane, while the ameloblasts showed vacuole-like changes in knockout mice (*n* = 3, Figure 2(b)). The HE staining clearly presented the damage of normal ameloblast polarity, with destruction of typical nucleus locating far from base membrane and apparent vacuole-like changes between the cells.

**Figure 3:**
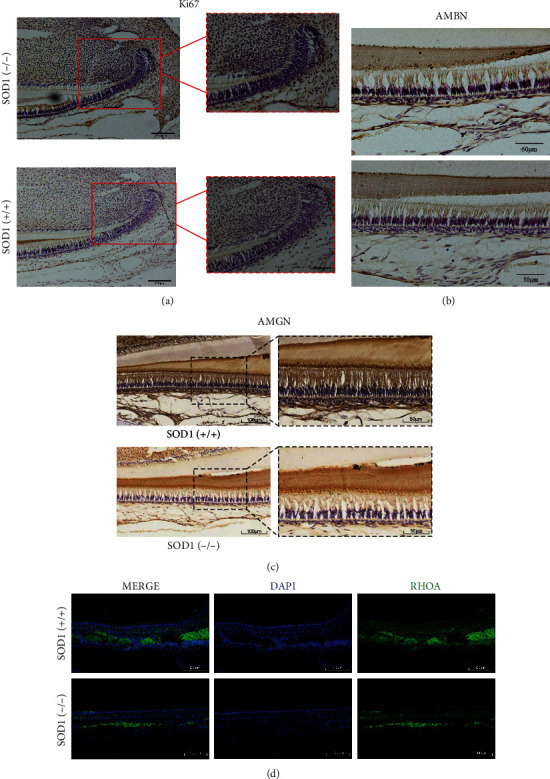
ROS accumulation changed Ki67 expression pattern and decreased RhoA and AMGN expressions. (a) The outer enamel epithelium of SOD1 (-/-) mice exhibited the greatest proliferation capacity, followed by the metastatic proliferation zone, and the stellate reticulum cells had a relatively moderate proliferation ability. (b–d) The difference of expression of AMBN, AMGN, and RhoA between SOD1 (-/-) mice and SOD1 (+/+) mice. Scale bars: (a–d) 100 *μ*m and 50 *μ*m; *n* = 3, respectively.

Ki67 is a recognized immunohistochemical marker associated with cell proliferation. The expression of ki67 in incisor cervical loop stem cells of SOD1 gene knockout mice indicated that the outer enamel epithelium had the strongest proliferation ability, followed by the metastatic proliferation zone, and the relatively weak proliferation ability was the stellate reticulum cells. But in the 4-month wild-type mice, the strongest proliferation ability belonged to the metastatic proliferation zone cells, and the proliferation ability of the inner and outer enamel epithelial cells and the stellate reticulum cells shared little difference. Compared with wild-type mice, the proliferation ability of incisor cervical loop stem cells was stronger in 4-month SOD1 knockout mice exhibiting more cells expressing ki67, whether in the inner and outer enamel epithelial layers, stellate reticulum layer, or the metastatic proliferation zone (*n* = 3, Figure 3(a)).

Acted as scaffolds for enamel crystals and secreted by ameloblasts, matrix proteins are closely related to the normal development of enamel. As the main enamel-forming protein, amelogenin constitutes nearly 90% organic matrix and mainly secreted by ameloblasts of the inner enamel epithelium during tooth development. Ameloblastin is another protein in the developing enamel matrix. Studies have shown that changes in its gene structure can cause several types of enamel hypoplasia. The expression of AMBN in ameloblasts derived from the epithelial stem cells of the incisor cervical loop at various stages showed that the expression of AMBN was found in preameloblasts, secretory ameloblasts, and mature ameloblasts. However, we found that there was no significant difference in the expression of AMBN in ameloblasts between two groups (*n* = 3, respectively, Figure 3(b)).

Tissue sections with positive RhoA protein, located in the cytoplasm, exhibited brownish yellow. It showed higher expression of AMGN protein in wild-type mice compared with the knockout group (*n* = 3, respectively, Figure 3(c)). The immunofluorescent staining of RhoA confirmed the disruption of ameloblast morphology and obvious discontinuities in the basement membrane (Figure 3(d)). All these sections revealed that SOD1-mediated ROS accumulation leads to the aberrant ameloblast-specific morphology and lower expression of AMGN and RhoA, suggesting that ameloblasts would impair under aberrant oxidative stress.

### 3.3. H_2_O_2_-Induced Oxidative Stress Had Dual Effects on Proliferation and Migration of Cervical Loop Stem Cells Depending on the Concentration of ROS

After 24 hours, the primary cells crawled out of the cell aggregate. The primary cells were a mixture of epithelial and mesenchymal cells that were connected tightly between each other. Polygonal epithelial stem cells grew in clusters, arranging in a typical paving stone shape, while mesenchymal stem cells were shaped in spindle-like cells. After repeated purification, almost all cells left were epithelial cells. Cytoimmunofluorescence detection found that CK14, the gold standard marker of epithelial cells, was almost 100% positive, indicating that the purified cells were almost epithelial cells. Sox2 is an experimentally verified and characteristic cell marker for cervical loop stem cells, and 90% of the epithelial stem cells we isolated and cultured were positively expressed. In addition, vimentin, a mesenchymal cell marker, stained negatively. HAT-7 cells were identified with CK14 and AMBN (Figure S2).

**Figure 4:**
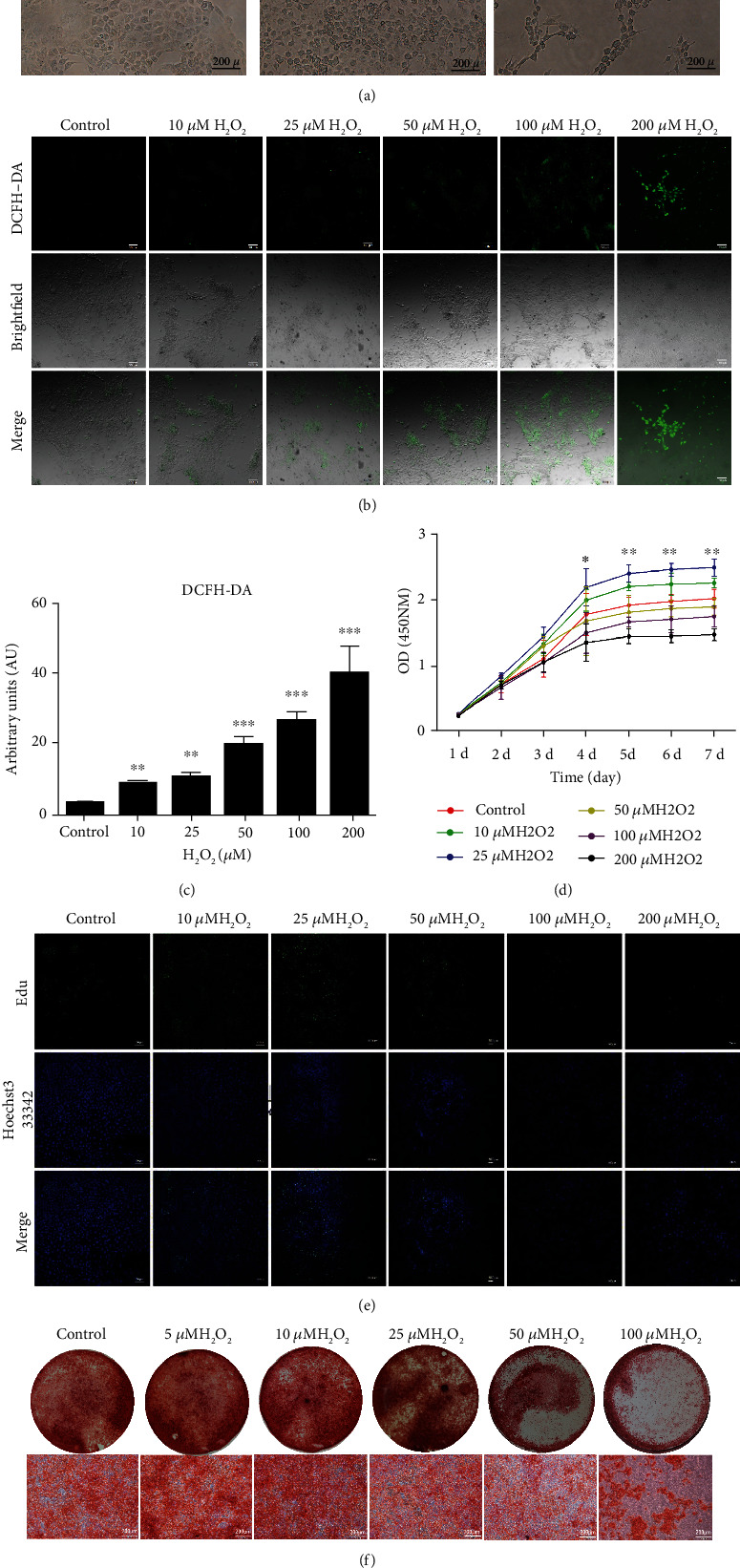
H_2_O_2_-induced oxidative stress exhibited dual effects on cervical loop stem cell proliferation and migration depending on the concentration of ROS. (a) The cell morphology changed under high concentration of H_2_O_2_ (100 *μ*M and 200 *μ*M). (b, c) The intracellular ROS concentration increased to approximately 2-2.5 times the normal concentration under 10 *μ*M-25 *μ*M H_2_O_2_. The intracellular ROS concentration increased to approximately 5-7 times the normal one under 50 *μ*M, 100 *μ*M, and 200 *μ*M H_2_O_2_. (d, e) 10 *μ*M-25 *μ*M H_2_O_2_ promoted proliferation while 50 *μ*M, 100 *μ*M, and 200 *μ*M H_2_O_2_ inhibited proliferation. 25 *μ*M H_2_O_2_ had the most obvious promoting effect. (f) 5 *μ*M and 10 *μ*M H_2_O_2_ promoted the differentiation and mineralization of incisor cervical loop epithelial stem cells, while 50 *μ*M and 100 *μ*M H_2_O_2_ had a significant inhibitory effect. Scale bars: (a, f) 200 *μ*m, (b, e) 100 *μ*m, and (a) 200 *μ*m. Data are shown as mean ± SD. ^∗^*P* < 0.05, ^∗∗^*P* < 0.01, and ^∗∗∗^*P* < 0.001.

Under the stimulation of low and medium concentrations of H_2_O_2_ (10 *μ*M-50 *μ*M), the cell morphology did not change significantly, but the effect of high concentration of H_2_O_2_ would cause the cell morphology to change. After stimulating with 100 *μ*M H_2_O_2_ for 6 hours, the cells lost their polygonal shape and became rounded and blunt, and there were several filamentous tentacle-like structures protruding from the periphery of the cells. After stimulating with 200 *μ*M H_2_O_2_ for 6 hours, cell adhesion decreased, and flakes were easy to fall off (Figure 4(a)). The DCFH-DA immunofluorescence probe is a universal oxidative stress indicator. The level of intracellular ROS is directly related to the fluorescence intensity. Observation by laser confocal fluorescence microscopy found that after stimulation with lower concentrations of H_2_O_2_ (10 *μ*M-25 *μ*M), the intracellular ROS concentration increased to approximately 2-2.5 times the normal concentration. Medium to high concentration (50 *μ*M-200 *μ*M) H_2_O_2_ stimulation caused the intracellular ROS concentration to increase to 5-7 times the normal concentration (Figure 4(b)).

CCK8 results showed that lower concentrations of H_2_O_2_ (10 *μ*M and 25 *μ*M H_2_O_2_) stimulated the proliferation of incisor cervical loop epithelial stem cells, of which 25 *μ*M H_2_O_2_ had the most obvious effect on cell proliferation, but higher concentrations of H_2_O_2_ (50 *μ*M, 100 *μ*M, and 200 *μ*M H_2_O_2_) inhibited cell proliferation, and the inhibitory effect was obvious with the increase of H_2_O_2_ concentration (^∗^*P* < 0.05, ^∗∗^*P* < 0.01, and ^∗∗∗^*P* < 0.001; Figures 4(c) and 4(d)). The EdU experiment further demonstrated the effect of short-term H_2_O_2_ stimulation (6 hours) on the proliferation of incisor cervical loop epithelial stem cells. The results showed consistence with the results of CCK-8 experiment, and lower concentration of H_2_O_2_ promoted proliferation while higher concentration of H_2_O_2_ inhibited proliferation. Among them, 25 *μ*M H_2_O_2_ had the most obvious promoting effect; 200 *μ*M H_2_O_2_ had the most significant inhibitory effect (Figure 4(e)).

Tooth enamel is a highly mineralized tissue mainly composed of crystalline calcium phosphate. The incisor cervical loop epithelial stem cells were maintained in a high-calcium differentiation medium for two weeks and then stained with Alizarin Red. The staining results showed that the incisor cervical loop epithelial stem cells were highly calcium-rich and produced calcified matrix material (Figure 4(f)). Alizarin Red staining results showed that the differentiation and mineralization ability of incisor cervical loop epithelial stem cells was more sensitive to changes of oxidative stress. 5 *μ*M H_2_O_2_ and10 *μ*M H_2_O_2_ promoted the differentiation and mineralization of incisor cervical loop epithelial stem cells. Multimineralized nodules could be seen in extracellular matrix under the microscope. 25 *μ*M H_2_O_2_ did not significantly promote or inhibit the differentiation and mineralization of incisor cervical loop epithelial stem cells; 50 *μ*M H_2_O_2_ had a significant inhibitory effect on the mineralization of incisor cervical loop epithelial stem cells.

### 3.4. ROS-Mediated Activation of PI3K-AKT in Cervical Loop Cell and Reduction of RhoA/ROCK Expression on Ameloblasts instead of Direct Detriment to Enamel Matrix Proteins

**Figure 5:**
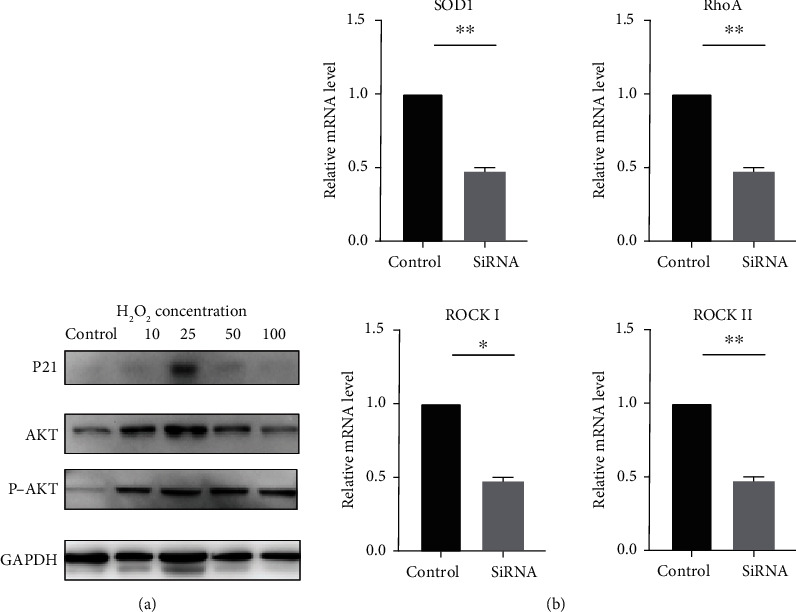
ROS-mediated activation of PI3K-AKT in cervical loop cell and reduction of RhoA/ROCK expression on ameloblasts instead of direct detriment to enamel matrix proteins. (a) The PI3K-AKT signaling pathway was activated by 10 *μ*M, 25 *μ*M, 50 *μ*M, and 100 *μ*M H_2_O_2_ and elevated the expression of phosphorylated AKT. (b) The downregulation of RhoA-ROCK expression when treated with SOD1 siRNA. Data are shown as mean ± SD. ^∗^*P* < 0.05 and ^∗∗^*P* < 0.01.

In order to further explore the related mechanism of the above phenomenon, it was found through Western blot that changes in the concentration of ROS caused differences in the expression of related molecules in the PI3K-AKT pathway (Figure 5(a)). P21 is a protein related to cell proliferation, exhibiting the highest expression under 25 *μ*M H_2_O_2_ stimulation. 10 *μ*M, 25 *μ*M, 50 *μ*M, and 100 *μ*M H_2_O_2_ all activated the PI3K-AKT signaling pathway and increased the expression of phosphorylated AKT. The effects of 10 *μ*M, 25 *μ*M, and 50 *μ*M H_2_O_2_ increased the total AKT expression. It showed that the proliferation of incisor cervical loop epithelial stem cells stimulated by different concentrations of H_2_O_2_ may be caused by the activation of the PI3K-AKT signaling pathway.

Enamel-related proteins and the RhoA-ROCK level were further analysed by qPCR considering their important roles played in the regulation of enamel formation (^∗^*P* < 0.05 and ^∗∗^*P* < 0.01; Figure 5(b)). The results showed a significant downregulation of the RhoA-ROCK expression when treated with SOD1 siRNA, indicating the underlying mechanism of impairment of ameloblast functions caused by ROS augmentation.

## 4. Discussion

Preliminary research results of our group showed that the level of H_2_O_2_ and oxidation product MDA in SOD1 knockout mice at the age of 4 months was higher than that of wild-type mice. Therefore, this experiment chose 4-month-old SOD1 knockout mice as the main experimental subjects [7]. As a model for studying ROS accumulation, SOD1 knockout mice have been proven to cause continuous and extensive oxidative damage, exhibiting liver cancer, amyotrophic lateral sclerosis, bone loss, and increased bone fragility [5, 28, 29].

Humans only replace their teeth once, while rodents show ever-growing incisors relying on epithelial stem cell niche. In the incisor CL, the loosely arranged reticular stellate epithelial cells are called the stellar reticular layer (SR) sandwiched between the inner enamel epithelium (IEE) and the outer enamel epithelium (OEE). Some of the cells in the SR are epithelial stem cells and proliferate into transport proliferation (TA) cells. These cells will differentiate along the TA into enamel-secreting ameloblasts [30, 31]. Our results showed that the accumulation of ROS caused by SOD1 gene knockout significantly changed the enamel structure of mouse incisors, leading to loss of enamel transparency; the cut ends were chalky, the enamel surface color disappeared, and it was easy to break. Scanning electron microscopy showed glaze pillar structure. The 4-month-old SOD1 knockout mice had aberrant tissue structure of the incisor cervical loop, but the number of proliferating cells was more than that of wild-type mice. Immunohistochemical staining showed that there was no significant difference in the expression of ameloblastin between SOD1 knockout mice and wild-type mice, while the expression of amelogenin decreased in transgenic mice. The aberrant development of enamel was usually interfered by different environmental influences and genetic changes, such as infection, trauma, changes in blood oxygen saturation, and tetracycline effects. In the process of enamel formation, the phenotype of the enamel caused by different types of damage will be different due to the type of interference factors and the time and intensity of action. Enamel defects can be divided into enamel quantity defects (hyperplasia) or insufficient mineral content (insufficient mineralization). The enamel phenotype of SOD1 knockout mice observed in our experiments may be related to insufficient enamel mineralization [32].

H_2_O_2_ stimulation of incisor cervical loop epithelial stem cells generated an increase in intracellular ROS. Higher ROS stimulation induced the cells to be round and blunt with filamentous tentacles and lower cellular adhesion. Lower ROS stimulation promoted the proliferation, short-term migration, and differentiation of incisor cervical loop epithelial stem cells, but higher concentration stimulation constrained the proliferation, migration, and differentiation of incisor cervical loop epithelial stem cells. This process was closely related to the activation of P-AKT and the increase of total AKT expression in the PI3K-AKT signaling pathway. The differentiation ability of incisor cervical loop epithelial stem cells was more sensitive to ROS, and the increase of ROS level obviously impeded their differentiation and mineralization ability. Stem cells respond to environmental signals that stimulate cell division in both stem cells and their multipotent offspring [33]. Several conserved diffuse signaling molecules have been proposed that function to regulate stem cell self-renewal. Western blot experiments also found that lower concentrations of ROS triggered the PI3K-AKT signaling pathway and boosted the expression of phosphorylated AKT, especially when stimulated by 25 *μ*M H_2_O_2_, the expression of phosphorylated AKT was the highest, and its proliferation effect was also the most noticeable. The possible mechanism of low concentration of ROS to promote the proliferation of incisor cervical loop epithelial stem cells is to activate the PI3K-AKT signaling pathway. AKT is the main regulator of the PI3K/AKT pathway, which regulates various cellular processes including cell growth, survival, proliferation, glucose metabolism, transcription, and protein synthesis [34]. Studies have shown that serine/threonine-specific protein kinases MAPK and AKT are both important effectors of growth factor receptor signaling pathways, and they are both directly mobilized by redox [35]. The basic mechanism of ROS-mediated redox signaling is located on specific redox-reactive amino acid residues. ROS-mediated oxidation of target proteins (also called redox sensors) is critical to protein function. Specifically, it is known that the sulfur-containing amino acids cysteine (Cys) and methionine (Met) are involved in redox signal transduction. In addition, transcription factors constitute an important class of ROS-responsive proteins, including NF-*κ*B, AP-1, H1F-1a, p53, and upstream stimulating factor (USF) [36]. The p21 protein is not only an inhibitor of the cell cycle but also an activator of the cell cycle, depending on the cell environment and its expression level. Studies have found that p21 leads to the inhibition of a variety of caspase and apoptotic effectors, including pro-caspase-3, caspase-8, caspase-10, apoptotic signal-regulated kinase 1, and stress-activated proteins kinase [37]. Our study found that only 25 *μ*M H_2_O_2_ stimulation increased the expression of P21 protein, which was significantly higher than that of the control group and other groups. The lower concentration of ROS promoted the proliferation of cervical loop stem cells, which confirmed the results of in vivo experiments. When the level of oxidative stress is higher than the physiological level, the proliferation of cervical loop stem cells increases, while their own differentiation and mineralization ability decreases, suggesting that as a stem cell, when the level of oxidative stress increases, it may be protecting itself as a stem cell pool instead of differentiation.

As a cell proliferation-related protein, p21 was significantly increased under 25 *μ*M H_2_O_2_ stimulation, compared with other groups. The p21 protein is not only an inhibitor of the cell cycle but also an activator of the cell cycle, depending on the cellular environment and its level of expression. Studies have found that p21 leads to the inhibition of multiple caspase proteases and apoptotic effectors, including pro-caspase-3, caspase-8, caspase-10, apoptosis signal-regulated kinase 1, and stress-activated protein kinase [37]. Our study found that only 25 *μ*M H_2_O_2_ stimulation increased P21 protein expression, a significantly higher level than the control and other groups. The results of the CCK-8 and the EdU cell proliferation experiment also showed that 25 *μ*M H_2_O_2_ had the most obvious effect on cell proliferation. Western blotting experiments also found that lower concentrations of hydrogen peroxide can activate the PI3K-AKT signaling pathway and increase the expression of phosphorylated AKT. Especially under the stimulation of 25 *μ*M H_2_O_2_, the expression of phosphorylated AKT is the highest, and its effect on promoting proliferation is also the most obvious, indicating that the possible mechanism of lower concentrations of hydrogen peroxide to promote the proliferation of incisor cervical ring epithelial stem cells is to activate the PI3K-AKT signaling pathway. AKT is a master regulator of the PI3K/AKT pathway, which regulates a variety of cellular processes, including cell growth, survival, proliferation, glucose metabolism, transcription, and protein synthesis [34].

The IF results indicated that there was a sharp drop of RhoA expression in mice with deprivation of SOD1, and the IHC results implied mild expression of amelogenin ameloblasts both in secretory and early maturation phase. Despite the unaffected expression of enamel matrix proteins, the RhoA and ROCK were downregulated under ROS accretion via SOD1 siRNA. In the secretory phase, ameloblasts form Tomes processes, the typical cell protrusions near the secretory cell membrane, which secrete enamel matrix proteins, such as amelogenin, ameloblastin, and enamelin to thicken enamel layers. With secretory and nonsecretory cell membrane structures on both sides, the Tomes process determines the conformational arrangement of enamel crystals and provides a scaffold structure for the further mineralization of enamel. With further differentiation, ameloblasts in the mature phase present secrete matrix protease such as KLK4 and MMP20 to degrade matrix protein for the mineralization of enamel. Amelogenin is expressed in the cytoplasm and enamel matrix of the secretory ameloblast. Apart from the cells of the stratum intermedium and stellate reticulum throughout amelogenesis, amelogenin was clearly observed in other phases including inner enamel epithelium of the cervical loop, secretory ameloblasts, and even those at the early maturation stage [38]. Its expression has a high degree of temporal and spatial specificity, suggesting that they are involved in enamel formation and mineralization [39]. Although in vitro experiment displayed consistent enamel matrix proteins in SOD1 siRNA-infected ameloblasts, immunochemistry sections confirmed a slight decline of amelogenin in secretory ameloblasts in SOD1 transgenic mice. Since HAT7 cell lines are dental epithelial cell line originating from the cervical loop of rat incisor and exhibit several ameloblast characteristics, they are commonly used as substitutes of ameloblasts in studying tooth development. HAT7 cell lines have been confirmed as functionally polarized like ameloblasts [40]. Several pathways including integrin-ODAM-ARHGEF5-RhoA and semaphorin 4D-RhoA-AKT participate in maintenance of ameloblast polarity and tight junctions. As a key factor, RhoA adjusts ameloblasts' secretion and differentiation with upstream effector like a bioswitch while it is still unclear about the contribution of its downstream molecule. Our results revealed that both RhoA and ROCK experienced downregulation under the high oxidative stress, which may be the crucial reason for the structure detriment of enamel in transgenic mice.

## 5. Conclusions

Taken together, our findings demonstrate the importance of ROS on pathological progress in enamel formation. Our results reveal the intricate effects of various levels of oxidation on the proliferation and differentiation of cervical loop cells. The results reported above provide further evidence for the importance of RhoA in amelogenesis. However, the present study has examined only one member in Rho GTPases. Therefore, more research is needed to investigate whether CDC42 and Rac 1 can also interact with RhoA under oxidative stress. We look forward to additional studies that offer new insight into the therapies of diseases related to enamel detriment in the clinic.

## Figures and Tables

**Table 1:** Primer sequences used for RT-PCR gene expression analysis.

Target cDNA	Primer sequence (5′-3′)
RhoA	GACCAGTTCCCAGAGGTTT
	CTGTGTCCCATAAAGCCAA

ROCKI	TATGAAGTAGTAAAGGTAATCGGCAGAG
	CTGGTGGATTTATGCCTTACCAA

ROCKII	AATCAAATCAGCATCCTTCTTTAAGAAT
	CTGGAGCTGCCGTCTCTCTTAT

SOD1	CAATGTGGCTGCTGGAA
	TGATGGAATGCTCTCCTGA

GAPDH	TATGACTCTACCCACGGCAAG
	TACTCAGCACCAGCATCACC

## Data Availability

The data used to support the findings of this study are available from the corresponding author upon request.

## Supplementary Materials

Supplementary Materials Supplementary Figure 1: identification of cervical loop cells and HAT-7 cells. The cervical loop primary cells are of mixed type with tight intercellular junctions, including epithelial stem cells that grow in clusters, which are polygonal and typical paving stones, and mesenchymal stem cells are spindle-shaped (a). Immunocytofluorescence detection showed that CK14, the gold standard marker of epithelial cells, was almost 100% positive, indicating that the purified cells were almost epithelial cells. Sox2 is an experimentally validated and characteristic cervical ring epithelial stem cell marker, which is positively expressed in 90% of our isolated and cultured epithelial stem cells. In addition, the mesenchymal cell marker vimentin stained negatively. HAT7 ameloblasts are regular polygons, with a typical cobblestone-like shape, the cells are closely arranged, the boundaries are clear, and the nucleus is obvious. At the same time, immunofluorescence staining of the cells showed that CK14 and ameloblastin (AMBN) were all positive, proving that they were epithelial-derived cells that secreted amelogenesis-related proteins (b).
