# Supplementary figures and images for: ROS-Mediated Enamel Formation Disturbance Characterized by Alternative Cervical Loop Cell Proliferation and Downregulation of RhoA/ROCK in Ameloblasts
Source: Oxid Med Cell Longev. 2022 Oct 17;2022:5769679. doi: 10.1155/2022/5769679 (PMC9592207; doi:10.1155/2022/5769679)

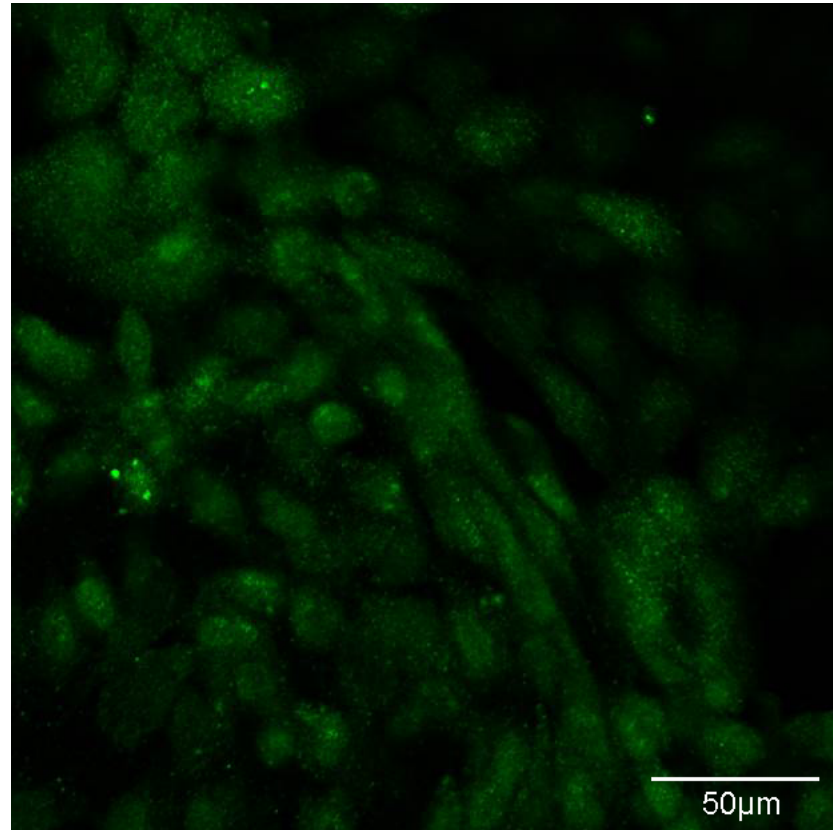

Supplement: Supplementary Materials — Supplementary Figure 1: identification of cervical loop cells and HAT-7 cells. The cervical loop primary cells are of mixed type with tight intercellular junctions, including epithelial stem cells that grow in clusters, which are polygonal and typical paving stones, and mesenchymal stem cells are spindle-shaped (a). Immunocytofluorescence detection showed that CK14, the gold standard marker of epithelial cells, was almost 100% positive, indicating that the purified cells were almost epithelial cells. Sox2 is an experimentally validated and characteristic cervical ring epithelial stem cell marker, which is positively expressed in 90% of our isolated and cultured epithelial stem cells. In addition, the mesenchymal cell marker vimentin stained negatively. HAT7 ameloblasts are regular polygons, with a typical cobblestone-like shape, the cells are closely arranged, the boundaries are clear, and the nucleus is obvious. At the same time, immunofluorescence staining of the cells showed that CK14 and ameloblastin (AMBN) were all positive, proving that they were epithelial-derived cells that secreted amelogenesis-related proteins (b). [file 5769679.f1.zip › AMBN.pdf]

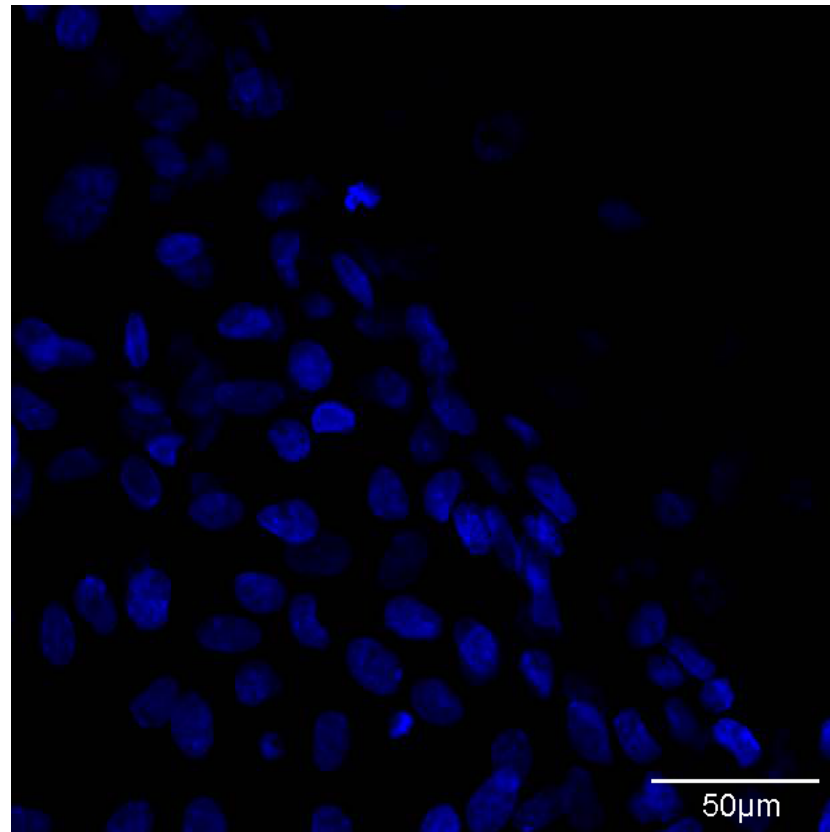

Supplement: Supplementary Materials — Supplementary Figure 1: identification of cervical loop cells and HAT-7 cells. The cervical loop primary cells are of mixed type with tight intercellular junctions, including epithelial stem cells that grow in clusters, which are polygonal and typical paving stones, and mesenchymal stem cells are spindle-shaped (a). Immunocytofluorescence detection showed that CK14, the gold standard marker of epithelial cells, was almost 100% positive, indicating that the purified cells were almost epithelial cells. Sox2 is an experimentally validated and characteristic cervical ring epithelial stem cell marker, which is positively expressed in 90% of our isolated and cultured epithelial stem cells. In addition, the mesenchymal cell marker vimentin stained negatively. HAT7 ameloblasts are regular polygons, with a typical cobblestone-like shape, the cells are closely arranged, the boundaries are clear, and the nucleus is obvious. At the same time, immunofluorescence staining of the cells showed that CK14 and ameloblastin (AMBN) were all positive, proving that they were epithelial-derived cells that secreted amelogenesis-related proteins (b). [file 5769679.f1.zip › AMBN-DAPI.pdf]

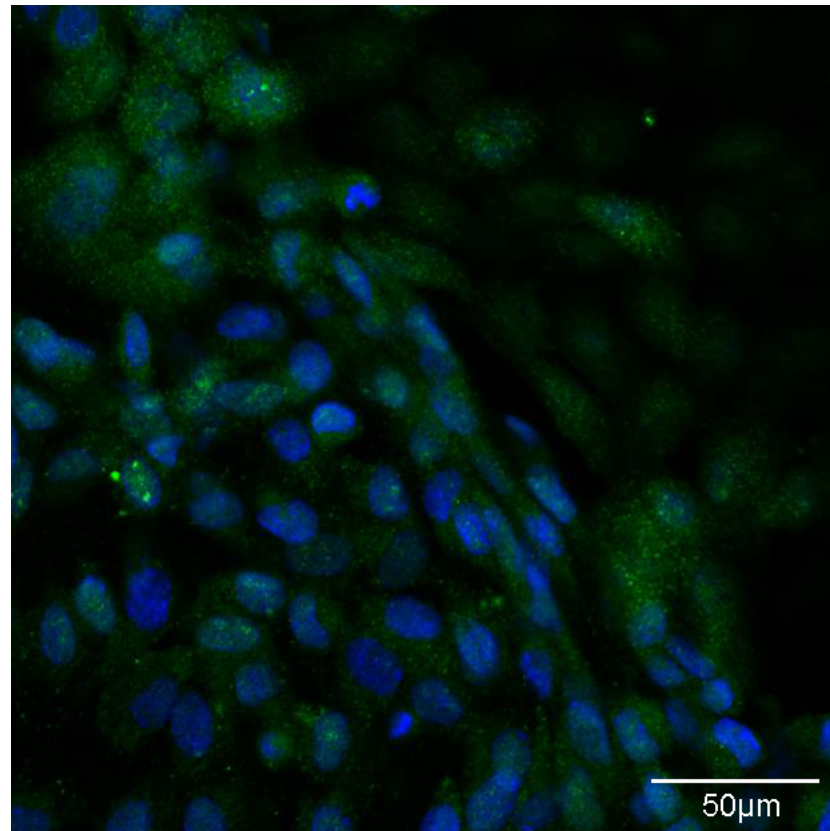

Supplement: Supplementary Materials — Supplementary Figure 1: identification of cervical loop cells and HAT-7 cells. The cervical loop primary cells are of mixed type with tight intercellular junctions, including epithelial stem cells that grow in clusters, which are polygonal and typical paving stones, and mesenchymal stem cells are spindle-shaped (a). Immunocytofluorescence detection showed that CK14, the gold standard marker of epithelial cells, was almost 100% positive, indicating that the purified cells were almost epithelial cells. Sox2 is an experimentally validated and characteristic cervical ring epithelial stem cell marker, which is positively expressed in 90% of our isolated and cultured epithelial stem cells. In addition, the mesenchymal cell marker vimentin stained negatively. HAT7 ameloblasts are regular polygons, with a typical cobblestone-like shape, the cells are closely arranged, the boundaries are clear, and the nucleus is obvious. At the same time, immunofluorescence staining of the cells showed that CK14 and ameloblastin (AMBN) were all positive, proving that they were epithelial-derived cells that secreted amelogenesis-related proteins (b). [file 5769679.f1.zip › AMBN-MERGE.pdf]

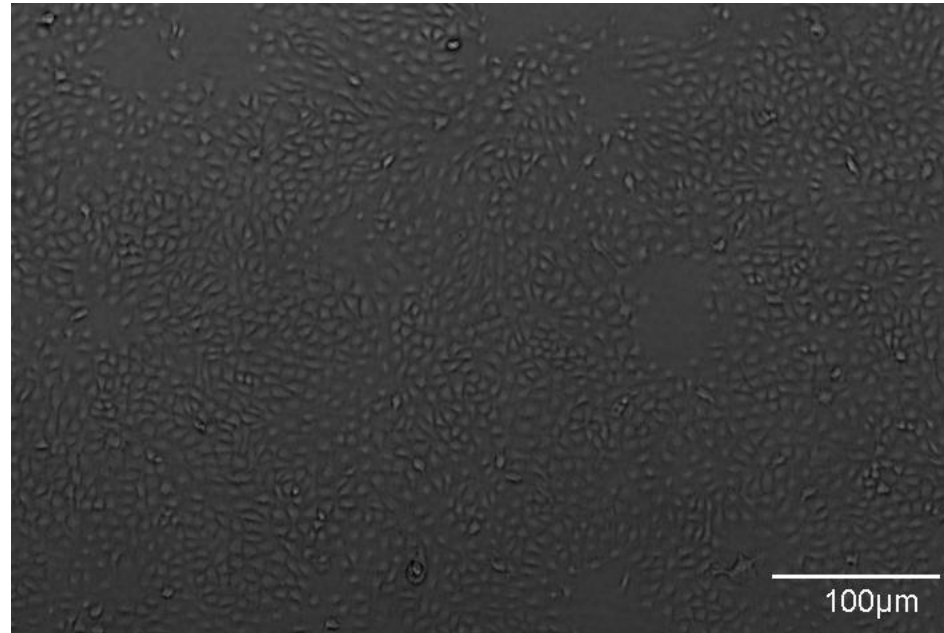

Supplement: Supplementary Materials — Supplementary Figure 1: identification of cervical loop cells and HAT-7 cells. The cervical loop primary cells are of mixed type with tight intercellular junctions, including epithelial stem cells that grow in clusters, which are polygonal and typical paving stones, and mesenchymal stem cells are spindle-shaped (a). Immunocytofluorescence detection showed that CK14, the gold standard marker of epithelial cells, was almost 100% positive, indicating that the purified cells were almost epithelial cells. Sox2 is an experimentally validated and characteristic cervical ring epithelial stem cell marker, which is positively expressed in 90% of our isolated and cultured epithelial stem cells. In addition, the mesenchymal cell marker vimentin stained negatively. HAT7 ameloblasts are regular polygons, with a typical cobblestone-like shape, the cells are closely arranged, the boundaries are clear, and the nucleus is obvious. At the same time, immunofluorescence staining of the cells showed that CK14 and ameloblastin (AMBN) were all positive, proving that they were epithelial-derived cells that secreted amelogenesis-related proteins (b). [file 5769679.f1.zip › ameloblasts.pdf]

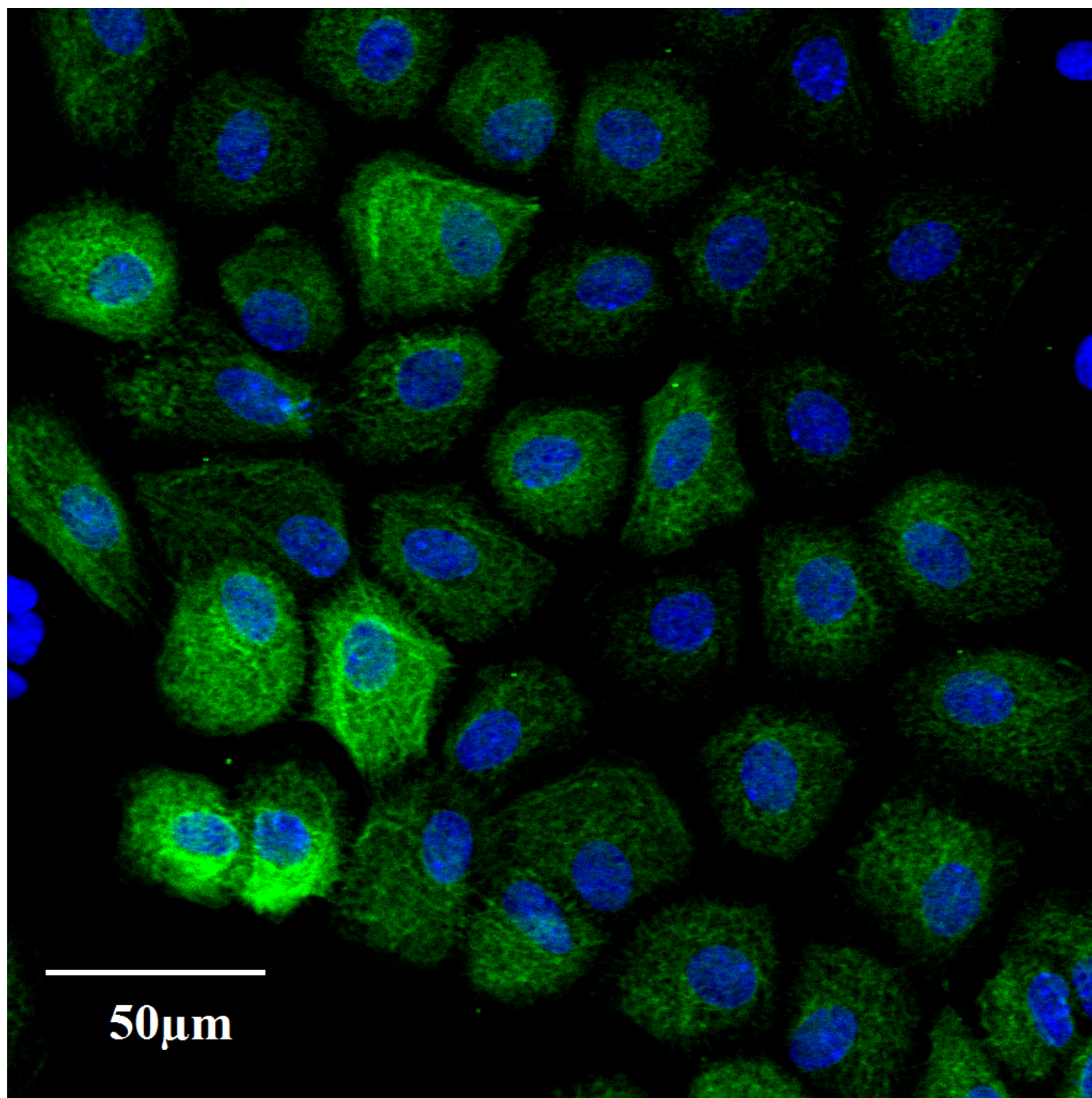

Supplement: Supplementary Materials — Supplementary Figure 1: identification of cervical loop cells and HAT-7 cells. The cervical loop primary cells are of mixed type with tight intercellular junctions, including epithelial stem cells that grow in clusters, which are polygonal and typical paving stones, and mesenchymal stem cells are spindle-shaped (a). Immunocytofluorescence detection showed that CK14, the gold standard marker of epithelial cells, was almost 100% positive, indicating that the purified cells were almost epithelial cells. Sox2 is an experimentally validated and characteristic cervical ring epithelial stem cell marker, which is positively expressed in 90% of our isolated and cultured epithelial stem cells. In addition, the mesenchymal cell marker vimentin stained negatively. HAT7 ameloblasts are regular polygons, with a typical cobblestone-like shape, the cells are closely arranged, the boundaries are clear, and the nucleus is obvious. At the same time, immunofluorescence staining of the cells showed that CK14 and ameloblastin (AMBN) were all positive, proving that they were epithelial-derived cells that secreted amelogenesis-related proteins (b). [file 5769679.f1.zip › ck14 40_.pdf]

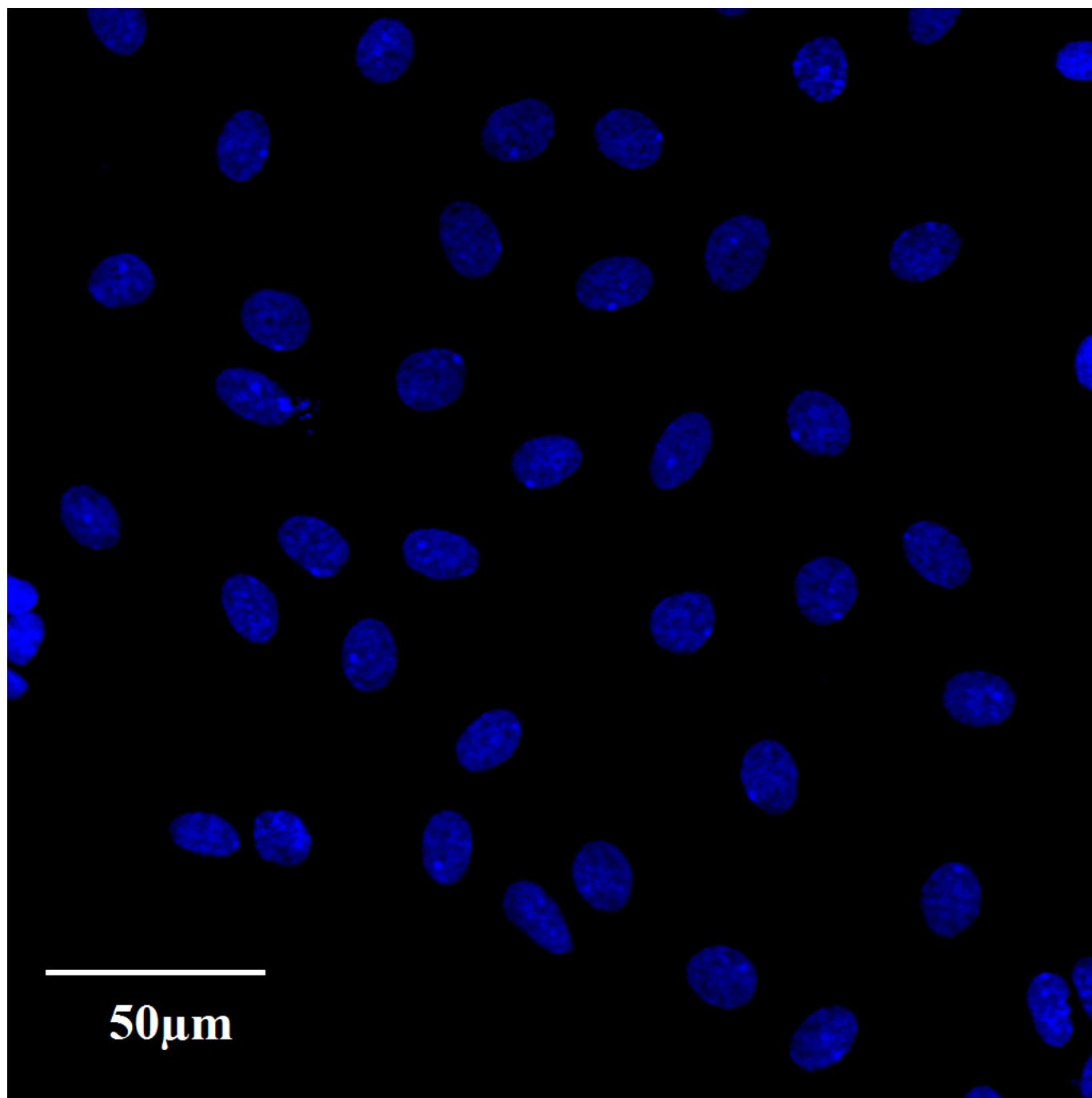

Supplement: Supplementary Materials — Supplementary Figure 1: identification of cervical loop cells and HAT-7 cells. The cervical loop primary cells are of mixed type with tight intercellular junctions, including epithelial stem cells that grow in clusters, which are polygonal and typical paving stones, and mesenchymal stem cells are spindle-shaped (a). Immunocytofluorescence detection showed that CK14, the gold standard marker of epithelial cells, was almost 100% positive, indicating that the purified cells were almost epithelial cells. Sox2 is an experimentally validated and characteristic cervical ring epithelial stem cell marker, which is positively expressed in 90% of our isolated and cultured epithelial stem cells. In addition, the mesenchymal cell marker vimentin stained negatively. HAT7 ameloblasts are regular polygons, with a typical cobblestone-like shape, the cells are closely arranged, the boundaries are clear, and the nucleus is obvious. At the same time, immunofluorescence staining of the cells showed that CK14 and ameloblastin (AMBN) were all positive, proving that they were epithelial-derived cells that secreted amelogenesis-related proteins (b). [file 5769679.f1.zip › ck14 40_C001.pdf]

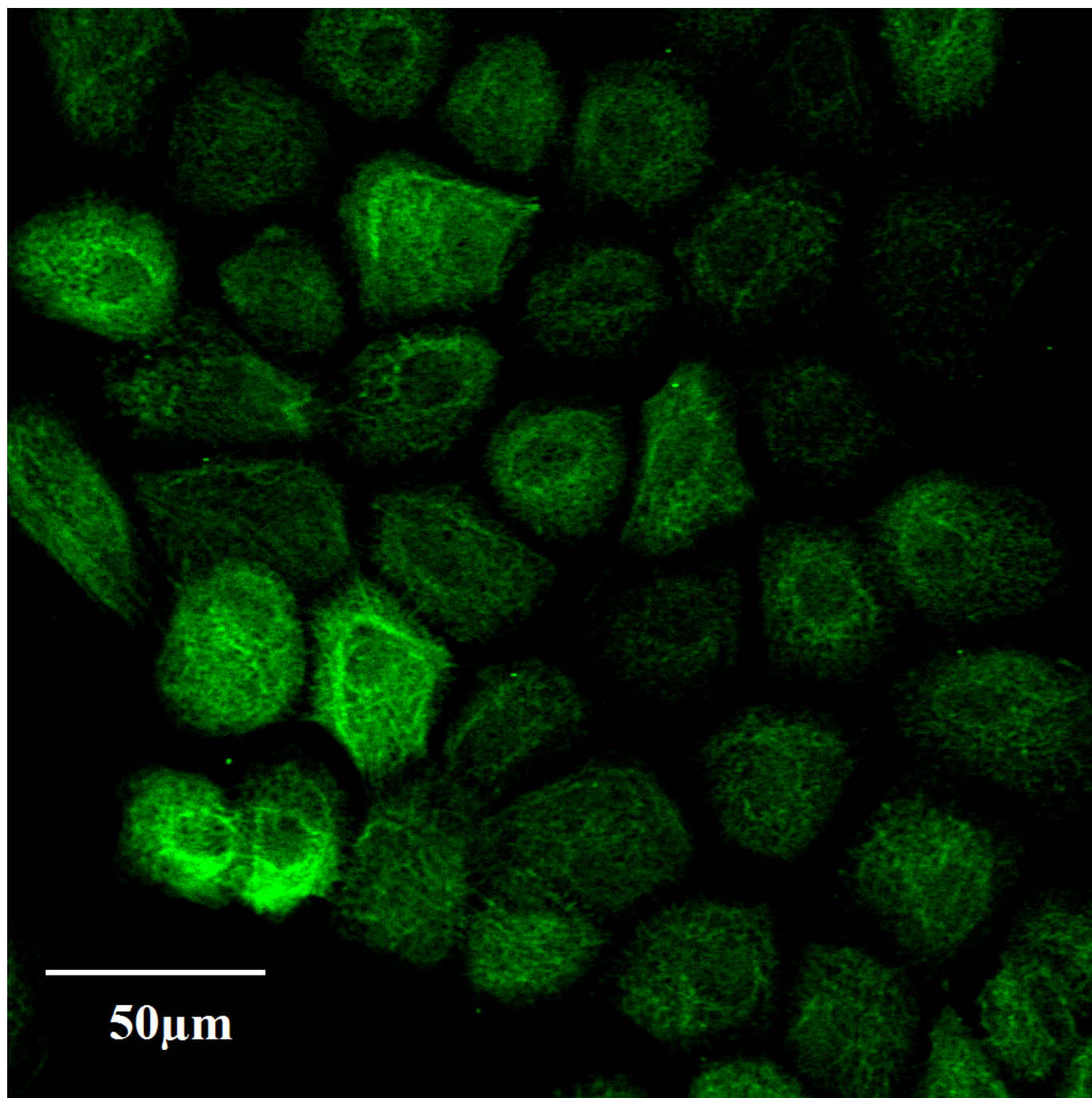

Supplement: Supplementary Materials — Supplementary Figure 1: identification of cervical loop cells and HAT-7 cells. The cervical loop primary cells are of mixed type with tight intercellular junctions, including epithelial stem cells that grow in clusters, which are polygonal and typical paving stones, and mesenchymal stem cells are spindle-shaped (a). Immunocytofluorescence detection showed that CK14, the gold standard marker of epithelial cells, was almost 100% positive, indicating that the purified cells were almost epithelial cells. Sox2 is an experimentally validated and characteristic cervical ring epithelial stem cell marker, which is positively expressed in 90% of our isolated and cultured epithelial stem cells. In addition, the mesenchymal cell marker vimentin stained negatively. HAT7 ameloblasts are regular polygons, with a typical cobblestone-like shape, the cells are closely arranged, the boundaries are clear, and the nucleus is obvious. At the same time, immunofluorescence staining of the cells showed that CK14 and ameloblastin (AMBN) were all positive, proving that they were epithelial-derived cells that secreted amelogenesis-related proteins (b). [file 5769679.f1.zip › ck14 40_C002.pdf]

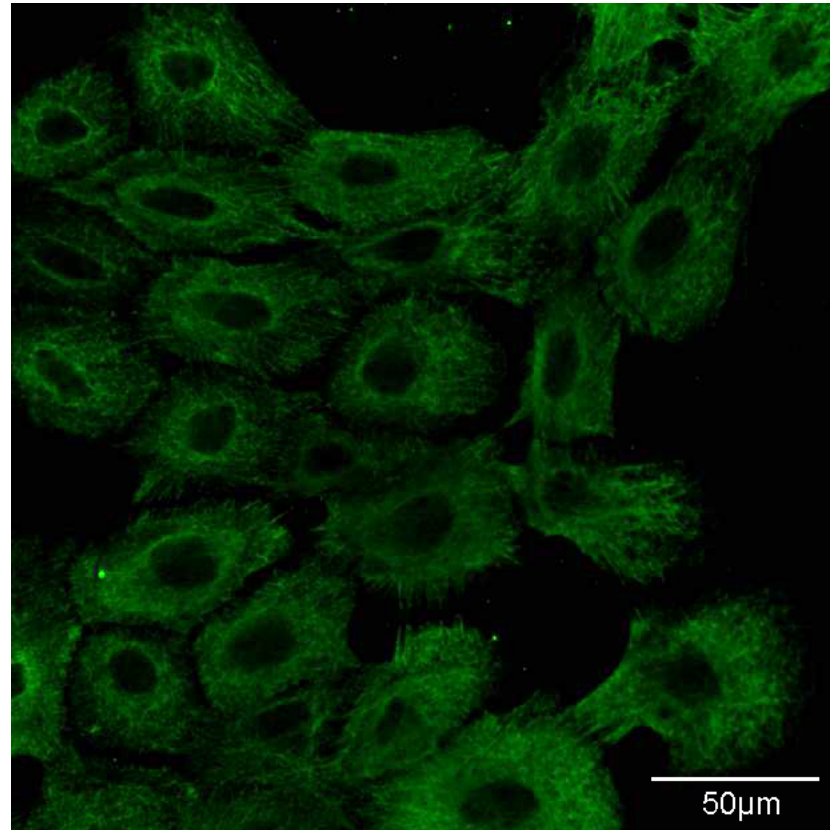

Supplement: Supplementary Materials — Supplementary Figure 1: identification of cervical loop cells and HAT-7 cells. The cervical loop primary cells are of mixed type with tight intercellular junctions, including epithelial stem cells that grow in clusters, which are polygonal and typical paving stones, and mesenchymal stem cells are spindle-shaped (a). Immunocytofluorescence detection showed that CK14, the gold standard marker of epithelial cells, was almost 100% positive, indicating that the purified cells were almost epithelial cells. Sox2 is an experimentally validated and characteristic cervical ring epithelial stem cell marker, which is positively expressed in 90% of our isolated and cultured epithelial stem cells. In addition, the mesenchymal cell marker vimentin stained negatively. HAT7 ameloblasts are regular polygons, with a typical cobblestone-like shape, the cells are closely arranged, the boundaries are clear, and the nucleus is obvious. At the same time, immunofluorescence staining of the cells showed that CK14 and ameloblastin (AMBN) were all positive, proving that they were epithelial-derived cells that secreted amelogenesis-related proteins (b). [file 5769679.f1.zip › CK14.pdf]

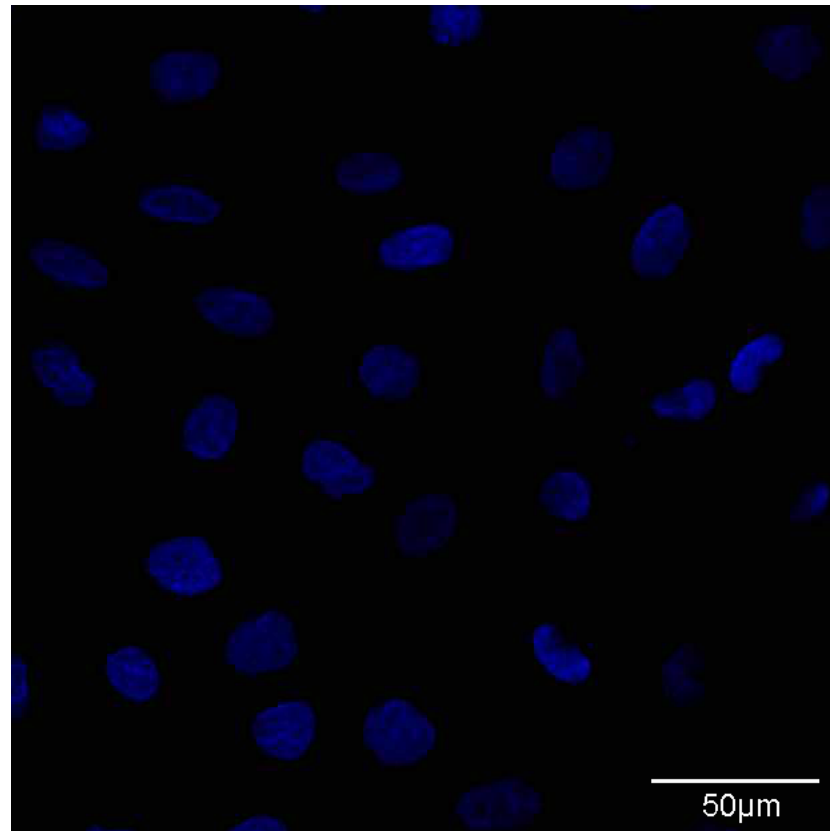

Supplement: Supplementary Materials — Supplementary Figure 1: identification of cervical loop cells and HAT-7 cells. The cervical loop primary cells are of mixed type with tight intercellular junctions, including epithelial stem cells that grow in clusters, which are polygonal and typical paving stones, and mesenchymal stem cells are spindle-shaped (a). Immunocytofluorescence detection showed that CK14, the gold standard marker of epithelial cells, was almost 100% positive, indicating that the purified cells were almost epithelial cells. Sox2 is an experimentally validated and characteristic cervical ring epithelial stem cell marker, which is positively expressed in 90% of our isolated and cultured epithelial stem cells. In addition, the mesenchymal cell marker vimentin stained negatively. HAT7 ameloblasts are regular polygons, with a typical cobblestone-like shape, the cells are closely arranged, the boundaries are clear, and the nucleus is obvious. At the same time, immunofluorescence staining of the cells showed that CK14 and ameloblastin (AMBN) were all positive, proving that they were epithelial-derived cells that secreted amelogenesis-related proteins (b). [file 5769679.f1.zip › CK14-dapi.pdf]

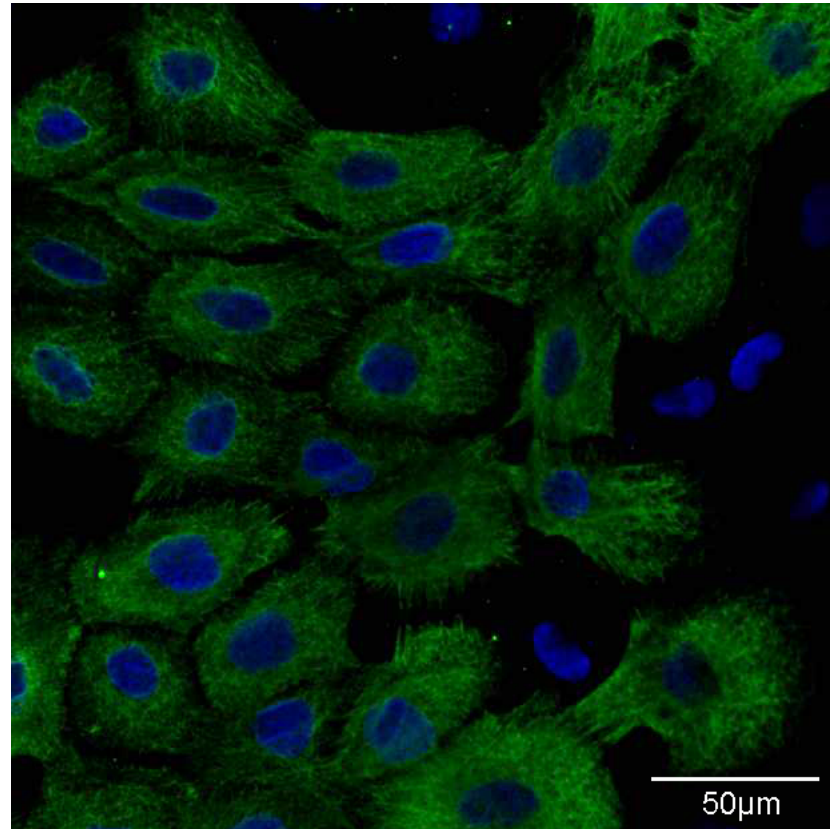

Supplement: Supplementary Materials — Supplementary Figure 1: identification of cervical loop cells and HAT-7 cells. The cervical loop primary cells are of mixed type with tight intercellular junctions, including epithelial stem cells that grow in clusters, which are polygonal and typical paving stones, and mesenchymal stem cells are spindle-shaped (a). Immunocytofluorescence detection showed that CK14, the gold standard marker of epithelial cells, was almost 100% positive, indicating that the purified cells were almost epithelial cells. Sox2 is an experimentally validated and characteristic cervical ring epithelial stem cell marker, which is positively expressed in 90% of our isolated and cultured epithelial stem cells. In addition, the mesenchymal cell marker vimentin stained negatively. HAT7 ameloblasts are regular polygons, with a typical cobblestone-like shape, the cells are closely arranged, the boundaries are clear, and the nucleus is obvious. At the same time, immunofluorescence staining of the cells showed that CK14 and ameloblastin (AMBN) were all positive, proving that they were epithelial-derived cells that secreted amelogenesis-related proteins (b). [file 5769679.f1.zip › CK14--merge.pdf]

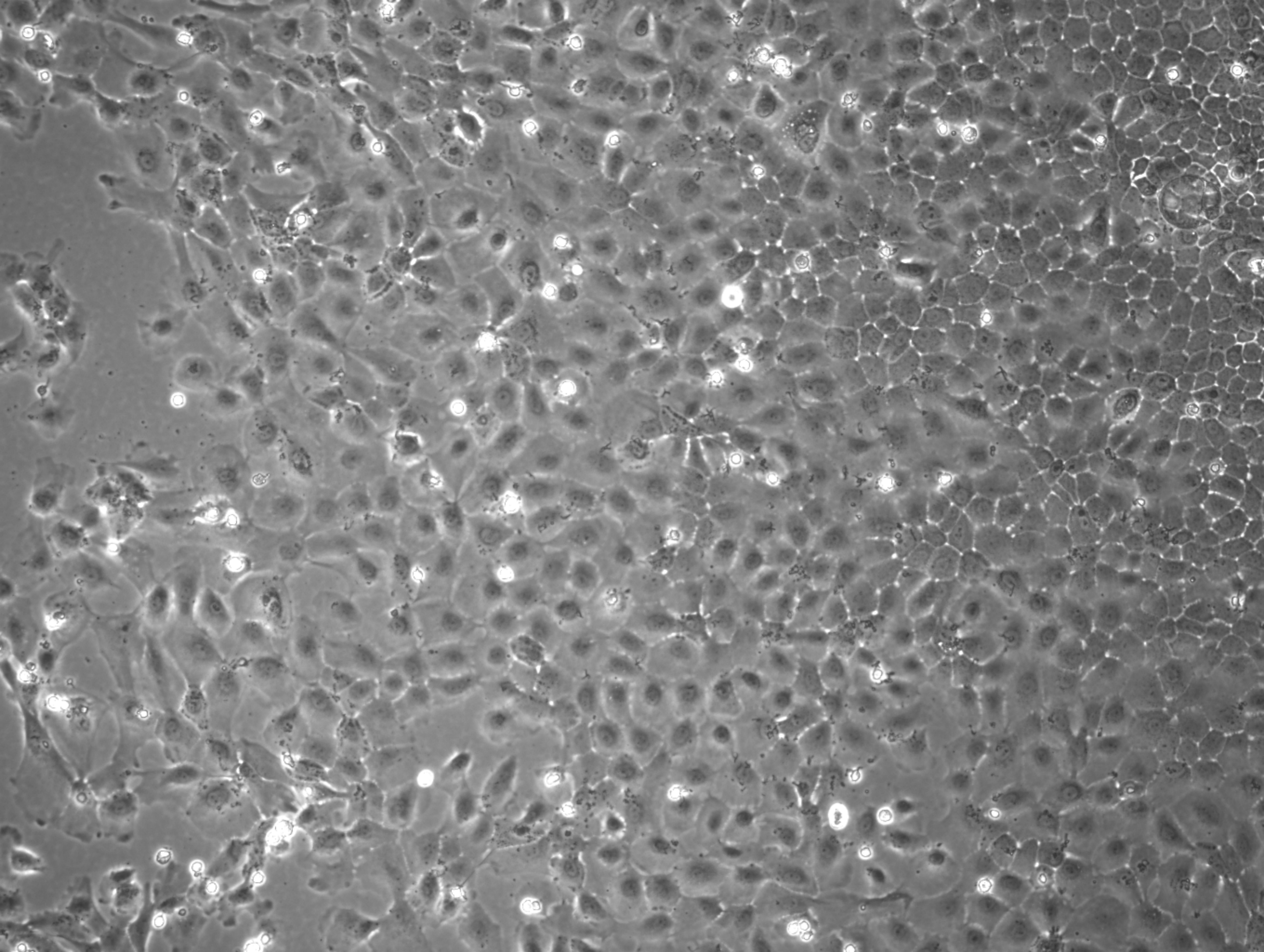

Supplement: Supplementary Materials — Supplementary Figure 1: identification of cervical loop cells and HAT-7 cells. The cervical loop primary cells are of mixed type with tight intercellular junctions, including epithelial stem cells that grow in clusters, which are polygonal and typical paving stones, and mesenchymal stem cells are spindle-shaped (a). Immunocytofluorescence detection showed that CK14, the gold standard marker of epithelial cells, was almost 100% positive, indicating that the purified cells were almost epithelial cells. Sox2 is an experimentally validated and characteristic cervical ring epithelial stem cell marker, which is positively expressed in 90% of our isolated and cultured epithelial stem cells. In addition, the mesenchymal cell marker vimentin stained negatively. HAT7 ameloblasts are regular polygons, with a typical cobblestone-like shape, the cells are closely arranged, the boundaries are clear, and the nucleus is obvious. At the same time, immunofluorescence staining of the cells showed that CK14 and ameloblastin (AMBN) were all positive, proving that they were epithelial-derived cells that secreted amelogenesis-related proteins (b). [file 5769679.f1.zip › FigS1-cervical loop cell.pdf]

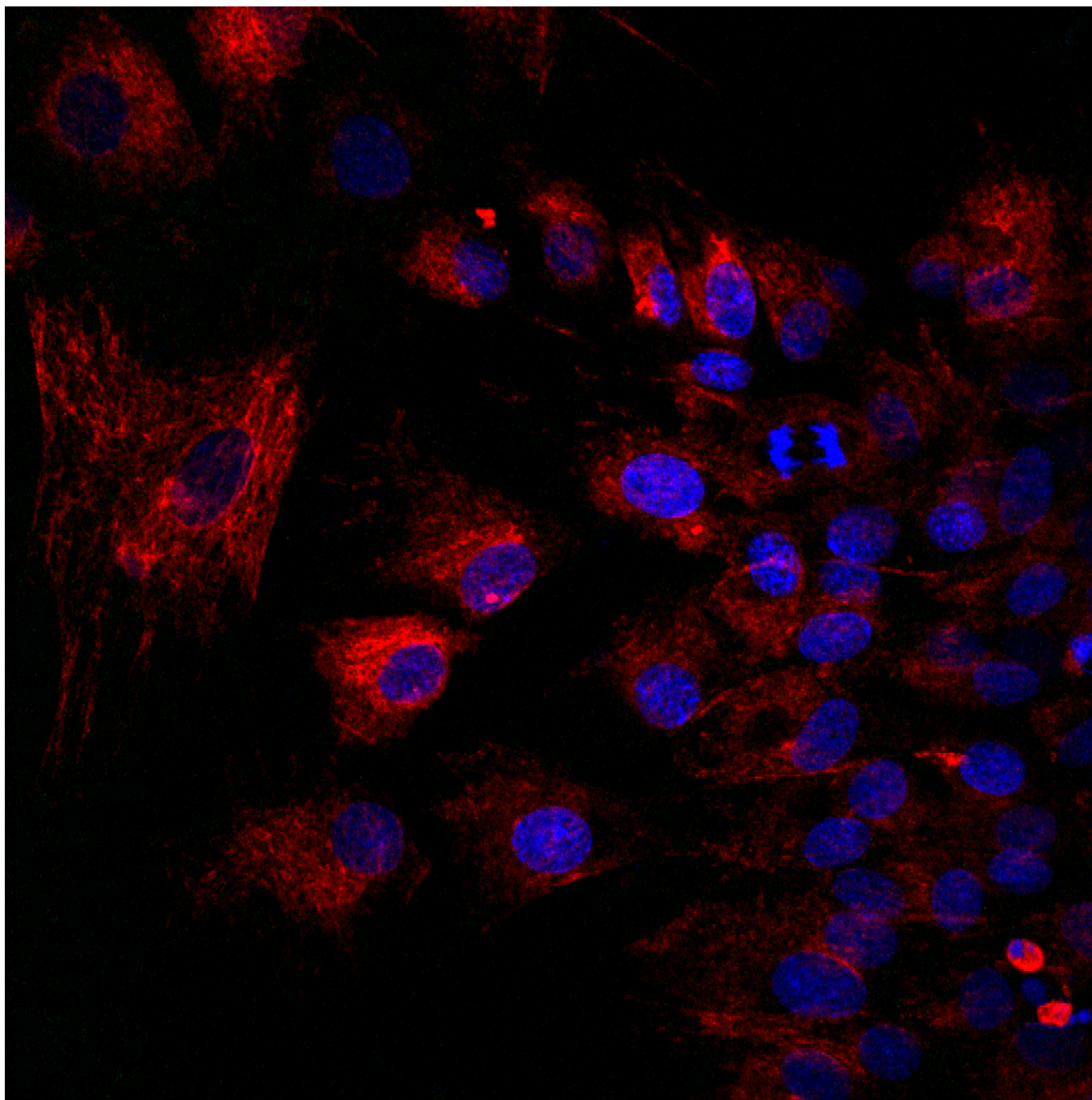

Supplement: Supplementary Materials — Supplementary Figure 1: identification of cervical loop cells and HAT-7 cells. The cervical loop primary cells are of mixed type with tight intercellular junctions, including epithelial stem cells that grow in clusters, which are polygonal and typical paving stones, and mesenchymal stem cells are spindle-shaped (a). Immunocytofluorescence detection showed that CK14, the gold standard marker of epithelial cells, was almost 100% positive, indicating that the purified cells were almost epithelial cells. Sox2 is an experimentally validated and characteristic cervical ring epithelial stem cell marker, which is positively expressed in 90% of our isolated and cultured epithelial stem cells. In addition, the mesenchymal cell marker vimentin stained negatively. HAT7 ameloblasts are regular polygons, with a typical cobblestone-like shape, the cells are closely arranged, the boundaries are clear, and the nucleus is obvious. At the same time, immunofluorescence staining of the cells showed that CK14 and ameloblastin (AMBN) were all positive, proving that they were epithelial-derived cells that secreted amelogenesis-related proteins (b). [file 5769679.f1.zip › sox2 60_.pdf]

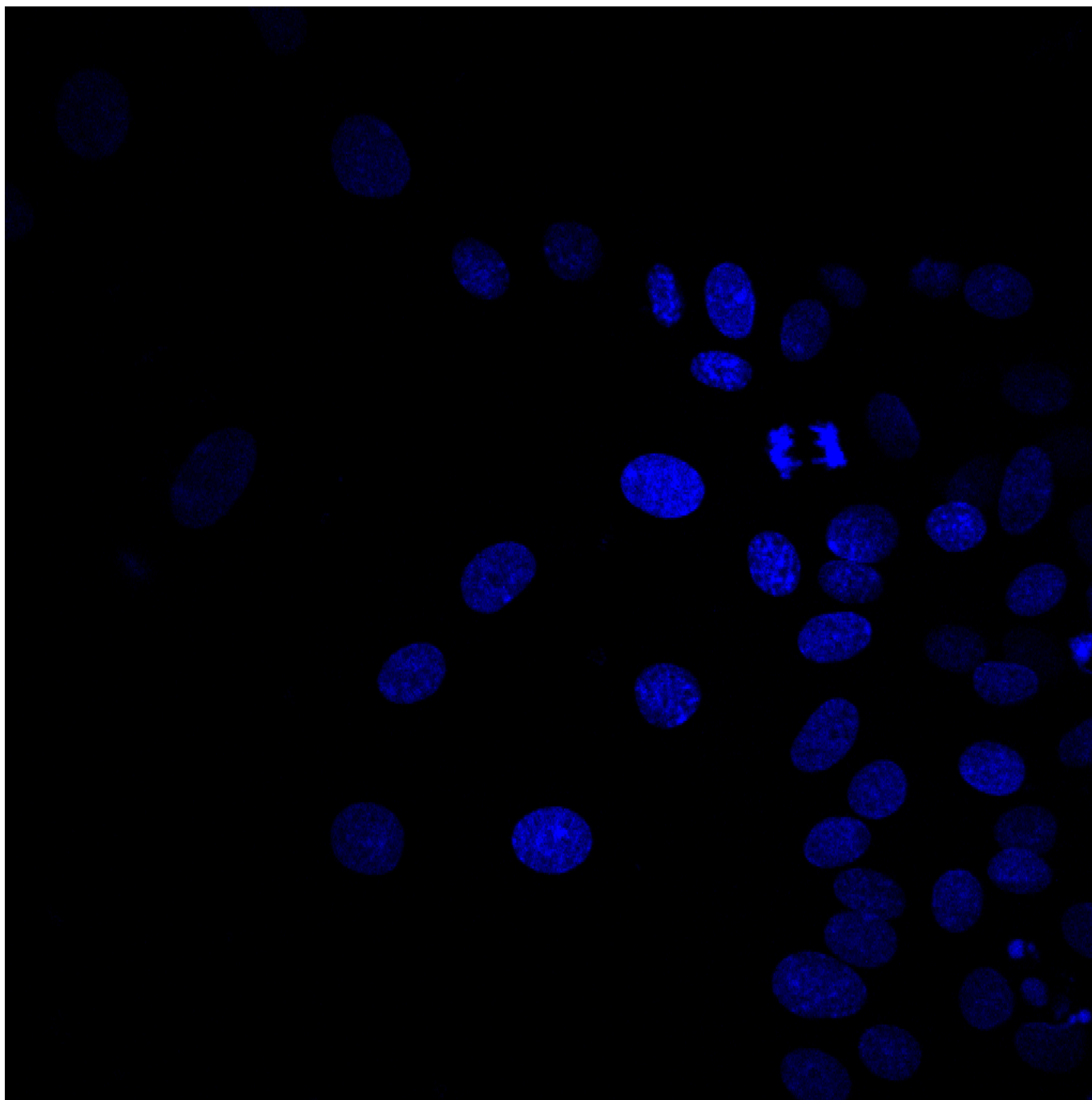

Supplement: Supplementary Materials — Supplementary Figure 1: identification of cervical loop cells and HAT-7 cells. The cervical loop primary cells are of mixed type with tight intercellular junctions, including epithelial stem cells that grow in clusters, which are polygonal and typical paving stones, and mesenchymal stem cells are spindle-shaped (a). Immunocytofluorescence detection showed that CK14, the gold standard marker of epithelial cells, was almost 100% positive, indicating that the purified cells were almost epithelial cells. Sox2 is an experimentally validated and characteristic cervical ring epithelial stem cell marker, which is positively expressed in 90% of our isolated and cultured epithelial stem cells. In addition, the mesenchymal cell marker vimentin stained negatively. HAT7 ameloblasts are regular polygons, with a typical cobblestone-like shape, the cells are closely arranged, the boundaries are clear, and the nucleus is obvious. At the same time, immunofluorescence staining of the cells showed that CK14 and ameloblastin (AMBN) were all positive, proving that they were epithelial-derived cells that secreted amelogenesis-related proteins (b). [file 5769679.f1.zip › sox2 60_C001.pdf]

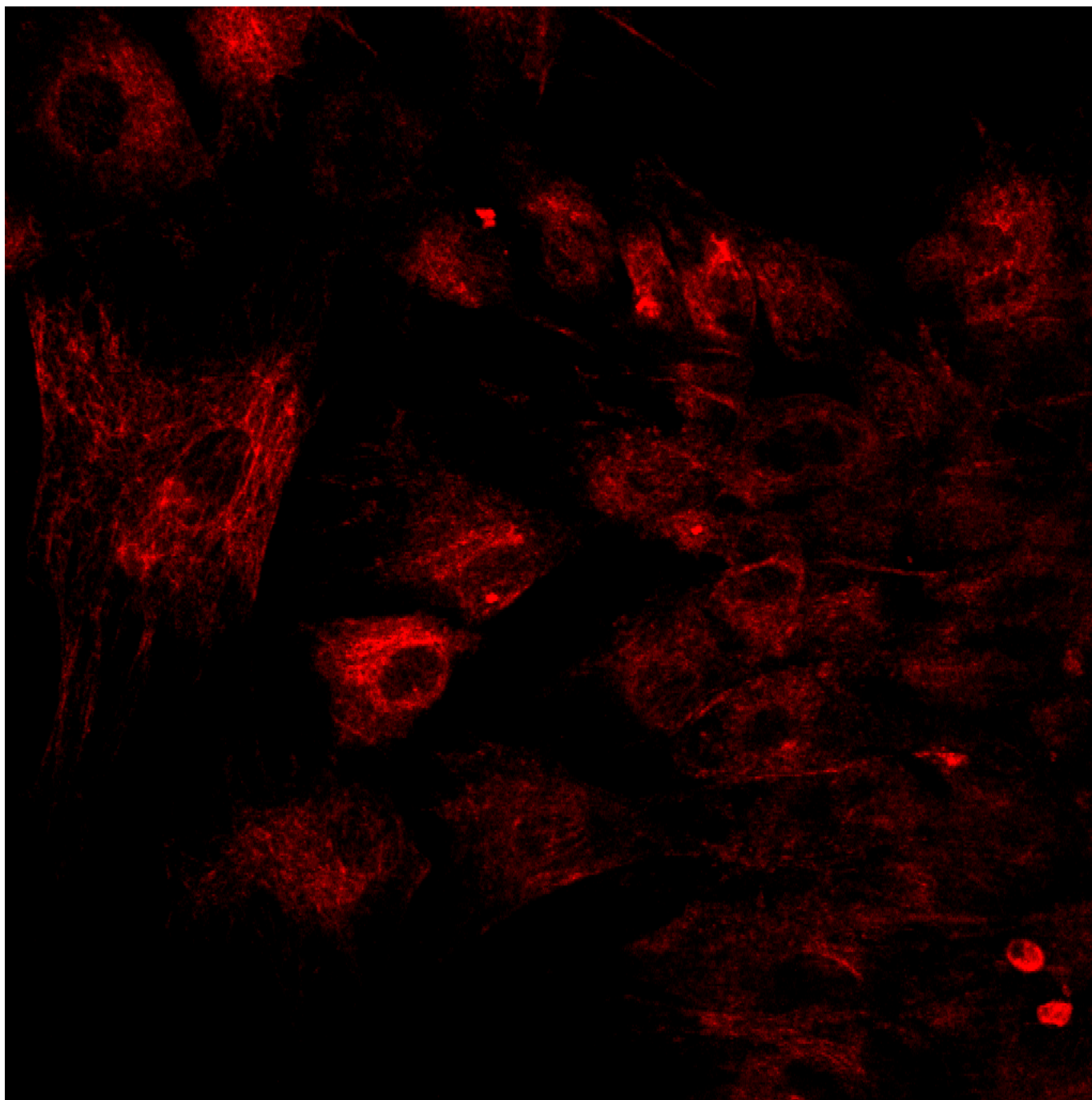

Supplement: Supplementary Materials — Supplementary Figure 1: identification of cervical loop cells and HAT-7 cells. The cervical loop primary cells are of mixed type with tight intercellular junctions, including epithelial stem cells that grow in clusters, which are polygonal and typical paving stones, and mesenchymal stem cells are spindle-shaped (a). Immunocytofluorescence detection showed that CK14, the gold standard marker of epithelial cells, was almost 100% positive, indicating that the purified cells were almost epithelial cells. Sox2 is an experimentally validated and characteristic cervical ring epithelial stem cell marker, which is positively expressed in 90% of our isolated and cultured epithelial stem cells. In addition, the mesenchymal cell marker vimentin stained negatively. HAT7 ameloblasts are regular polygons, with a typical cobblestone-like shape, the cells are closely arranged, the boundaries are clear, and the nucleus is obvious. At the same time, immunofluorescence staining of the cells showed that CK14 and ameloblastin (AMBN) were all positive, proving that they were epithelial-derived cells that secreted amelogenesis-related proteins (b). [file 5769679.f1.zip › sox2 60_C003.pdf]

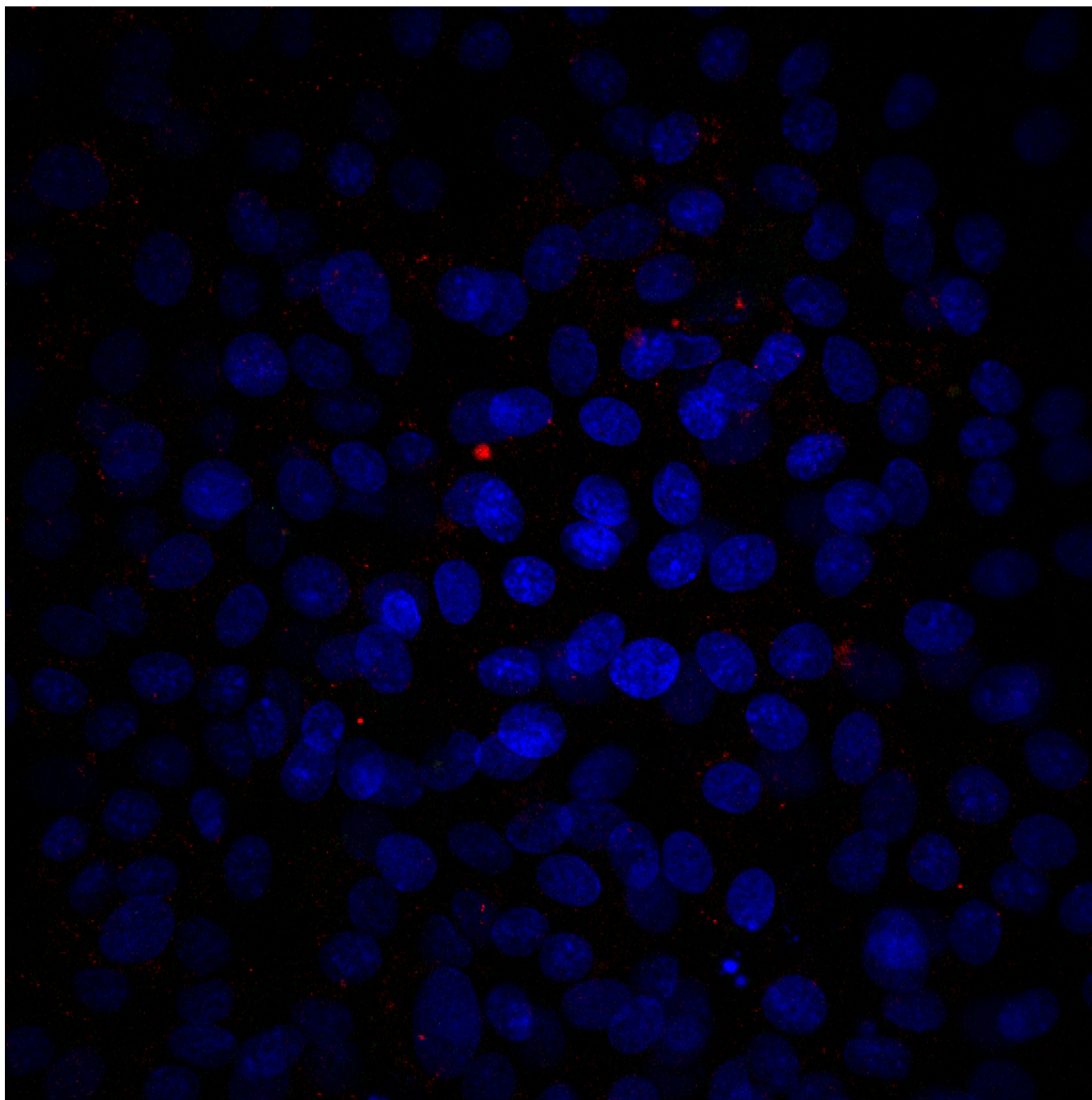

Supplement: Supplementary Materials — Supplementary Figure 1: identification of cervical loop cells and HAT-7 cells. The cervical loop primary cells are of mixed type with tight intercellular junctions, including epithelial stem cells that grow in clusters, which are polygonal and typical paving stones, and mesenchymal stem cells are spindle-shaped (a). Immunocytofluorescence detection showed that CK14, the gold standard marker of epithelial cells, was almost 100% positive, indicating that the purified cells were almost epithelial cells. Sox2 is an experimentally validated and characteristic cervical ring epithelial stem cell marker, which is positively expressed in 90% of our isolated and cultured epithelial stem cells. In addition, the mesenchymal cell marker vimentin stained negatively. HAT7 ameloblasts are regular polygons, with a typical cobblestone-like shape, the cells are closely arranged, the boundaries are clear, and the nucleus is obvious. At the same time, immunofluorescence staining of the cells showed that CK14 and ameloblastin (AMBN) were all positive, proving that they were epithelial-derived cells that secreted amelogenesis-related proteins (b). [file 5769679.f1.zip › viminten.pdf]

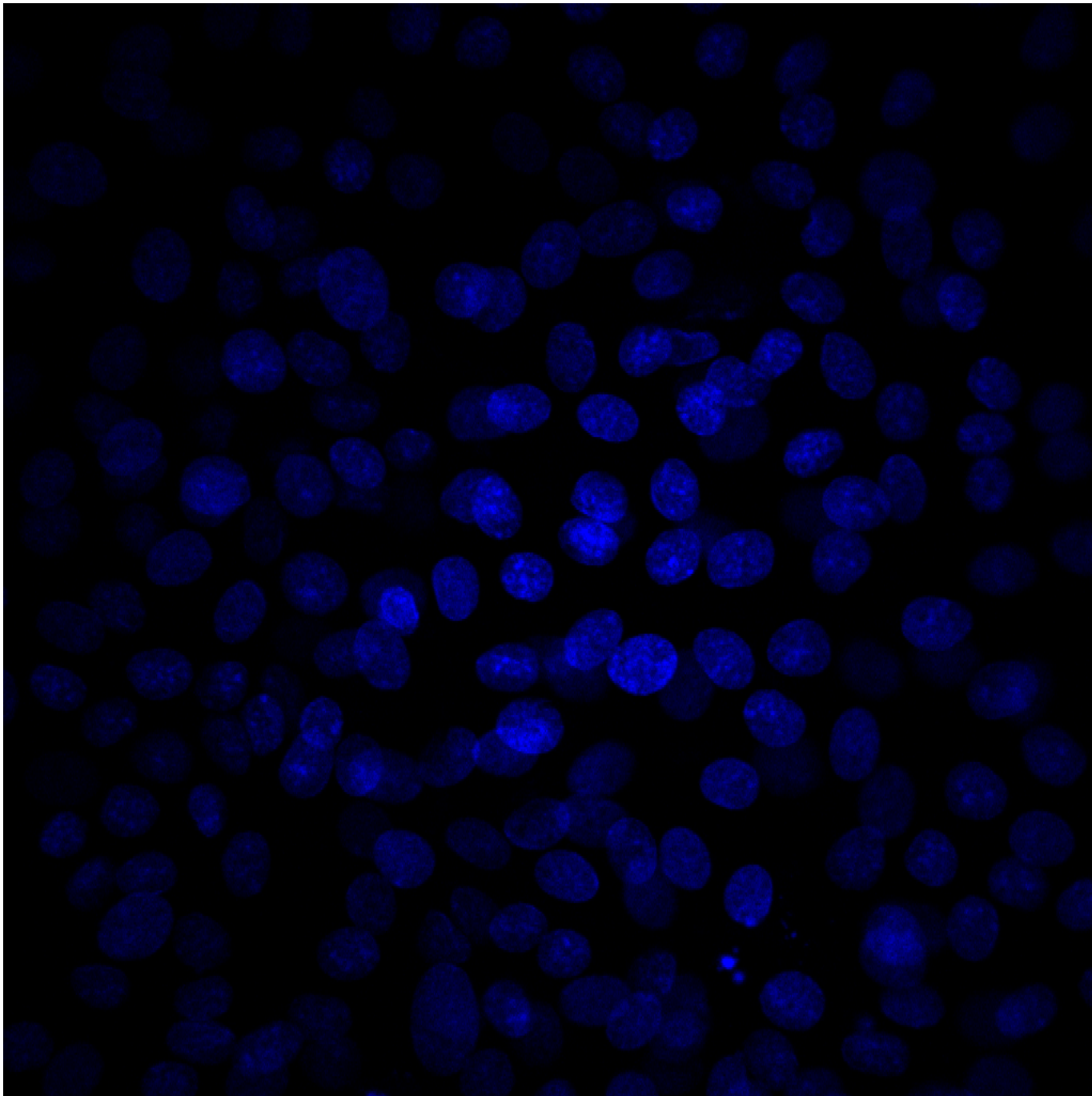

Supplement: Supplementary Materials — Supplementary Figure 1: identification of cervical loop cells and HAT-7 cells. The cervical loop primary cells are of mixed type with tight intercellular junctions, including epithelial stem cells that grow in clusters, which are polygonal and typical paving stones, and mesenchymal stem cells are spindle-shaped (a). Immunocytofluorescence detection showed that CK14, the gold standard marker of epithelial cells, was almost 100% positive, indicating that the purified cells were almost epithelial cells. Sox2 is an experimentally validated and characteristic cervical ring epithelial stem cell marker, which is positively expressed in 90% of our isolated and cultured epithelial stem cells. In addition, the mesenchymal cell marker vimentin stained negatively. HAT7 ameloblasts are regular polygons, with a typical cobblestone-like shape, the cells are closely arranged, the boundaries are clear, and the nucleus is obvious. At the same time, immunofluorescence staining of the cells showed that CK14 and ameloblastin (AMBN) were all positive, proving that they were epithelial-derived cells that secreted amelogenesis-related proteins (b). [file 5769679.f1.zip › viminten_C001.pdf]

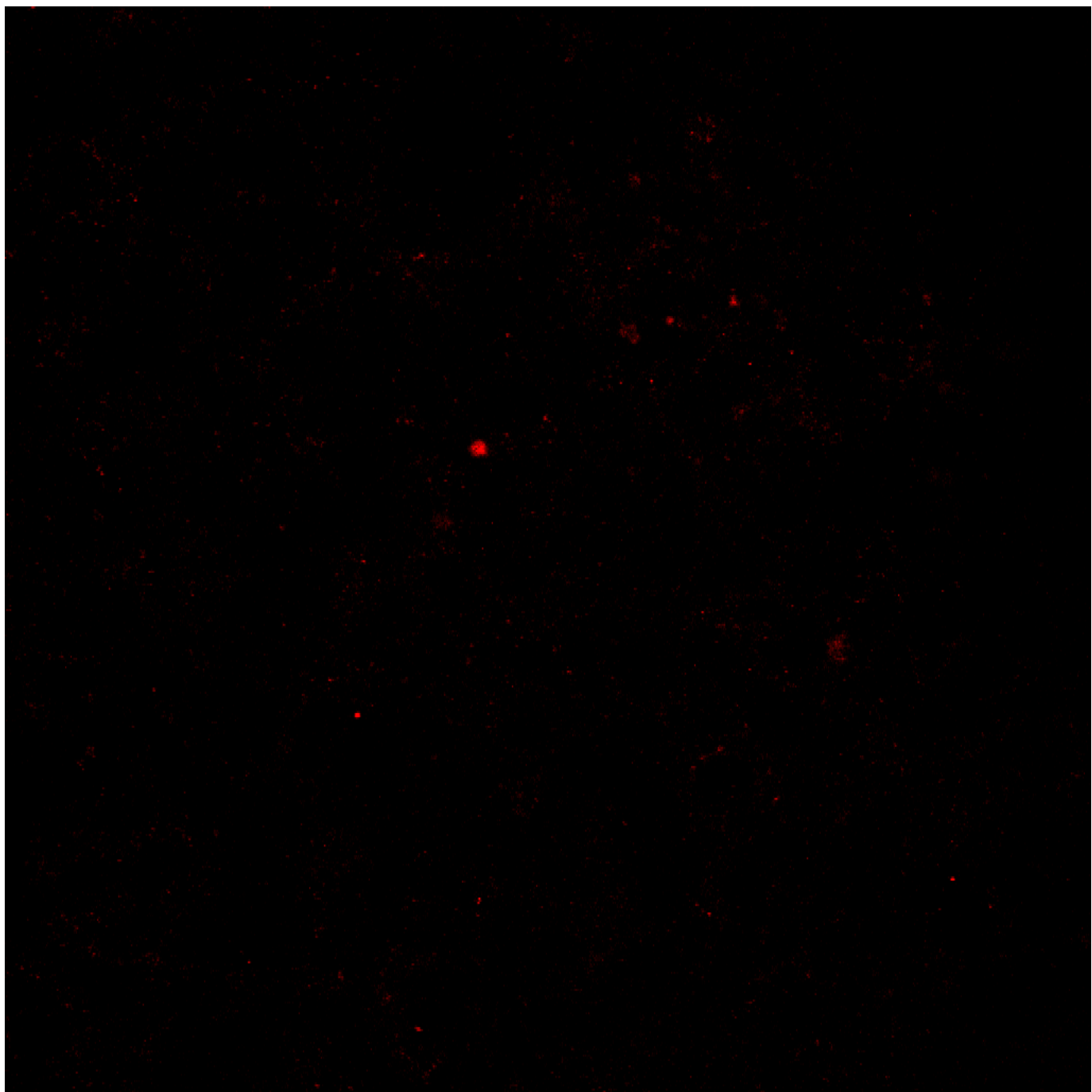

Supplement: Supplementary Materials — Supplementary Figure 1: identification of cervical loop cells and HAT-7 cells. The cervical loop primary cells are of mixed type with tight intercellular junctions, including epithelial stem cells that grow in clusters, which are polygonal and typical paving stones, and mesenchymal stem cells are spindle-shaped (a). Immunocytofluorescence detection showed that CK14, the gold standard marker of epithelial cells, was almost 100% positive, indicating that the purified cells were almost epithelial cells. Sox2 is an experimentally validated and characteristic cervical ring epithelial stem cell marker, which is positively expressed in 90% of our isolated and cultured epithelial stem cells. In addition, the mesenchymal cell marker vimentin stained negatively. HAT7 ameloblasts are regular polygons, with a typical cobblestone-like shape, the cells are closely arranged, the boundaries are clear, and the nucleus is obvious. At the same time, immunofluorescence staining of the cells showed that CK14 and ameloblastin (AMBN) were all positive, proving that they were epithelial-derived cells that secreted amelogenesis-related proteins (b). [file 5769679.f1.zip › viminten_C003.pdf]
